# Cholesterol Overload Drives Hepatic Steatosis by Inhibiting OGT-dependent PPARα O-GlcNAcylation and Transactivation

**DOI:** 10.7150/ijbs.135054

**Published:** 2026-05-18

**Authors:** Rui Guo, Yanhui Li, Qinchao Ding, Chad Slawson, Udayan Apte, Yuwei Jiang, Xiaobing Dou, Songtao Li, Zhenyuan Song

**Affiliations:** 1 Department of Kinesiology and Nutrition, University of Illinois Chicago, Chicago, IL, USA.; 2 School of Public Health, Zhejiang Chinese Medical University, Hangzhou, Zhejiang, PR. China.; 3 Department of Biochemistry and Molecular Biology, Kansas University Medical Center, Kansas City, KS, USA; 4 Department of Pharmacology, Toxicology & Therapeutics, Kansas University Medical Center, Kansas City, KS, USA; 5 Department of Physiology & Biophysics, University of Illinois Chicago, Chicago, IL, USA; 6 School of Life Science, Zhejiang Chinese Medical University, Hangzhou, Zhejiang, PR. China.

**Keywords:** hepatic steatosis, cholesterol, O-GlcNAcylation, OGT, PPARα

## Abstract

Although dietary cholesterol is known to exacerbate liver disease progression, whether and how it contributes to hepatic steatosis, the hallmark early pathological feature of both MASLD and ALD, remains poorly understood. Here, we investigated how cholesterol disrupts hepatic triacylglycerol metabolism using both dietary and cellular cholesterol-loading models. Integrated transcriptomic, metabolomic, and biochemical analyses were performed, and causality was examined through genetic and pharmacologic modulation in multiple hepatocyte systems and mice. Our results demonstrate that cholesterol overload induces hepatocellular fat accumulation in a dose-dependent, cell-autonomous manner, primarily by suppressing fatty acid β-oxidation. Mechanistically, we identified PPARα inhibition as a key event underlying this effect. Cholesterol overload suppressed PPARα transactivation, thereby impairing fatty acid β-oxidation and promoting hepatocellular fat accumulation. This inhibition was mechanistically linked to reduced O-GlcNAcylation. Specifically, cholesterol overload downregulated OGT, leading to reduced protein O-GlcNAcylation and consequent PPARα inhibition; similarly, liver-specific OGT knockout mice exhibited suppressed PPARα activity and increased hepatic fat accumulation. RNA-sequencing and co-immunoprecipitation analyses identified PPARα as an O-GlcNAc-modified protein, and loss of this modification impaired its transactivity. Functionally, restoration of O-GlcNAcylation via genetic OGA knockdown or pharmacological activation of PPARα with WY14643 alleviated cholesterol-induced hepatic steatosis in mice without altering hepatic cholesterol levels. Lastly, we identified SREBP2 as the upstream transcriptional regulator linking cholesterol overload to OGT suppression. In conclusion, our findings in this study uncover a previously unrecognized cholesterol-OGT-PPARα axis that suppresses hepatic fatty acid β-oxidation and drives steatosis. Targeting O-GlcNAc cycling or activating PPARα represents a promising therapeutic strategy for MASLD.

## Introduction

Metabolic dysfunction-associated steatotic liver disease (MASLD) has become the most common chronic liver disorder worldwide, encompassing a spectrum from simple steatosis to steatohepatitis, fibrosis, and cirrhosis 1-3. Its rising prevalence parallels increasing rates of obesity, insulin resistance, and Western dietary habits 4-6. Among dietary factors, cholesterol has emerged as an important contributor to MASLD progression. A growing body of clinical and experimental evidence demonstrates that cholesterol-enriched diets exacerbate hepatic inflammation, injury, and fibrogenic response, thereby accelerating MASLD progression 7-9. Intriguingly, despite its well-established role in promoting disease progression, it remains unclear whether and how cholesterol drives hepatic steatosis, the defining feature of early-stage MASLD, particularly given that cholesterol itself is not a direct substrate for triacylglycerol synthesis.

Peroxisome proliferator-activated receptor alpha (PPARα) is a ligand-activated nuclear receptor abundantly expressed in hepatocytes, where it orchestrates transcriptional programs that regulate lipid catabolism and energy homeostasis 10-12. Upon activation, PPARα induces genes involved in mitochondrial and peroxisomal β-oxidation, ketogenesis, and lipoprotein metabolism, thereby facilitating fatty acid clearance and maintaining hepatic metabolic integrity 13-16. In MASLD, reduced PPARα activity is a consistent molecular feature associated with hepatic steatosis, inflammation, and disease progression 11, 15, 17-19. Experimental evidence indicates that genetic ablation or pharmacological inhibition of PPARα suppresses fatty acid oxidation, promotes lipid accumulation, and increases susceptibility to diet-induced liver injury 19-21. Conversely, activation with selective agonists such as WY14643 or fenofibrate alleviates steatosis, inflammation, and fibrotic remodeling, underscoring the pivotal role of PPARα in protecting against MASLD pathogenesis 22-25. Protein O-GlcNAcylation is a dynamic and reversible post-translational modification in which O-linked N-acetylglucosamine (O-GlcNAc) is added to serine or threonine residues of nuclear and cytoplasmic proteins 26-28. This process is catalyzed by O-GlcNAc transferase (OGT) and removed by O-GlcNAcase (OGA), using UDP-GlcNAc generated through the hexosamine biosynthetic pathway (HBP) as the donor substrate 27, 29. Because HBP integrates inputs from glucose, glutamine, acetyl-CoA, and nucleotide metabolism, O-GlcNAcylation functions as a nutrient-sensing mechanism that couples cellular metabolic status to signaling and transcriptional regulation 30, 31. Accumulating evidence supports that dysregulated hepatic O-GlcNAcylation contributes to the development of multiple liver diseases, including MASLD, steatohepatitis, fibrosis, and hepatocellular carcinoma 32-35; however, current findings remain contradictory. On the one hand, several studies have reported that a high-fat diet (HFD) or metabolic overload elevates hepatic protein O-GlcNAcylation, either by enhancing *de novo* lipogenesis or by upregulating CD36, thereby accelerating the progression from MASLD to steatohepatitis (MASH) 30, 36-38. On the other hand, it has been clearly demonstrated that hepatocyte-specific OGT knockout mice exhibit profound metabolic and structural abnormalities, including increased liver injury, inflammation, fibrosis, and exacerbated development of DEN-induced hepatocellular carcinoma 39, 40. These opposing observations suggest that basal O-GlcNAcylation is essential for maintaining hepatic homeostasis and function, underscoring the complex and context-dependent role of O-GlcNAc signaling in liver metabolism. Therefore, it is imperative to clarify how nutrient or lipid overload, including cholesterol loading, modulates OGT-mediated O-GlcNAcylation and contributes to the initiation and progression of MASLD.

To elucidate whether and how cholesterol loading affects hepatocellular lipid metabolism, we employed integrated *in vitro* and *in vivo* approaches and identified a previously unrecognized cholesterol-OGT-PPARα signaling axis that mechanistically links free cholesterol accumulation to hepatocellular fat accumulation. We demonstrate that cholesterol overload induces hepatic steatosis by inhibiting PPARα activity via suppression of OGT-dependent protein O-GlcNAcylation. Free cholesterol, but not cholesteryl esters, downregulates OGT expression, leading to reduced global O-GlcNAcylation, including modification of PPARα, thereby impairing its transcriptional control of fatty acid β-oxidation and promoting lipid accumulation. Importantly, restoring O-GlcNAcylation or activating PPARα reverses cholesterol-induced steatosis, establishing a mechanistic cholesterol-OGT-O-GlcNAc-PPARα axis that represents a targetable pathway in MASLD development.

## Materials and Methods

### Reagents

Chemicals, including bovine serum albumin (BSA, A7030) and DMSO (D2650), were purchased from Sigma-Aldrich. Other chemicals used in this study were purchased as follows: Simvastatin (Adipogen, AG-CN2-0052), Low density lipoprotein (ThermoFisher, L3486), Cholesterol-water soluble (MCE, HY-N0322A), Etomoxir (MCE, HY-50202A), Pirinixic acid (MCE, HY-16995), Fenofibrate (MCE, HY-17356), Methyl-β-cyclodextrin (MCE, HY-101461), OSMI-1 (MCE, HY-119738), Thiamet G (MCE, HY-12588), Protein A/G PLUS-Agarose (Santa Cruz, sc-2003), Normal mouse IgG (Santa Cruz, sc-2025), and NP-40 alternative (Santa Cruz, CAS 9016-45-9).

### Cell culture

Murine AML12 and human HepG2 hepatocyte cell lines were obtained from the American Type Culture Collection (ATCC, VA, USA). Human hepatoma HepaRG cells (Thermo Fisher Scientific, HPRGC10), which are terminally differentiated and non-proliferative, were also used in this study. (See **supplementary information** for details).

### RNA interference

Cultured cells were transfected with *Ogt* siRNA (Invitrogen, 173150), *Soat2* (Invitrogen, 101666), and *Srebf2* (Invitrogen, 284040) using Lipofectamine 2000 (Invitrogen, 3016547) based on the manufacturer's instructions. Cells in the control group were transfected with scrambled siRNA (Santa Cruz, sc-37007). (See **supplementary information** for details).

### Fatty acid oxidation (FAO) staining

FAO activity was measured using the FAOBlue^TM^ detection reagent (Funakoshi, FDV-0033). (See **supplementary information** for details).

### Bodipy staining

Cells (1 × 10^5^) were seeded into 24-well plates and cultured for 16 hours. After treatment, cells were washed with PBS, fixed in 4% formaldehyde (Pierce, Rockford, IL) for 15 min, and rinsed again. 5 μM BODIPY 493/503 (Invitrogen, D3922) was applied for 15 min at room temperature. Following PBS washes, nuclei were counterstained with DAPI mounting medium (Sigma-rich, Saint Louis, MO). Images were captured using a LEICA inverted fluorescence microscope.

### Animal experiments

All mice were housed under specific pathogen-free (SPF) conditions, with controlled temperatures (18-23 °C), humidity (40-60%), and a 12-hour light/dark cycle. Mice had free access to food and water throughout the study. All animal studies were approved by the Institutional Animal Care and Use Committee at the University of Illinois Chicago and were conducted in accordance with the NIH Guide for the Care and Use of Laboratory Animals. Ten-week-old male C57BL/6 mice were used for dietary interventions with varying cholesterol concentrations (Sigma Grade, ≥99%, Sigma-Aldrich, C8667) and for pharmacological treatment with the PPARα agonist WY14643. In addition, ten-week-old male C57BL/6 mice were subjected to hepatic OGA knockdown using adeno-associated virus serotype 8 (AAV8)-mediated shRNA delivery. Mice received either AAV8-sh*Oga* or AAV8-shScrmb control via orbital injection during dietary intervention on day 10. Animals were maintained on a 2% cholesterol diet for 30 days after viral administration. (See **supplementary information** for details).

### Histological analysis

Liver tissues were fixed in 10% neutral-buffered formalin for 24 hours. Selected samples were embedded in paraffin, sectioned at 4 μM, and stained with hematoxylin and eosin (H&E) for histological evaluation. To assess collagen deposition, paraffin-embedded liver sections were stained with Sirius Red (G-Clone, Beijing, China) according to standard protocols. For lipid staining, portions of the liver were embedded in Tissue-Tek OCT compound (Sakura, Tokyo, Japan), frozen, sectioned at 8 μM, and stained with Oil Red O. Images were captured by a Zeiss Axio Observer A1 inverted microscope (Oberkochen, Germany).

### Biochemical assays

Hepatic and plasma triacylglycerol (TAG) and total cholesterol (TC) contents, as well as cellular lipid levels, were quantified using standard biochemical assays with commercial reagent kits (Jiancheng, Nanjing, China) according to the manufacturers' instructions. Cellular TAG and TC levels were normalized to protein content. (See **supplementary information** for details).

### Chromatin immunoprecipitation analysis (ChIP)

ChIP assays were performed using the Pierce^™^ Agarose ChIP Kit (Thermo Fisher, Rockford, IL, USA) according to the manufacturer's protocol. HepG2 cells were cultured in 6-well plates and treated with vehicle (UT), 4 μM cholesterol, or 10 μM simvastatin for 12 hours. Cells (~2 × 10^6^ per condition) were crosslinked in culture by adding formaldehyde to a final concentration of 1% for 10 min at room temperature, and the reaction was terminated with glycine. Chromatin was enzymatically fragmented and immunoprecipitated with an antibody specific to SREBF2. DNA was purified from immunoprecipitants and analyzed by quantitative PCR. The target region encompasses the Ogt gene promoter on chromosome X, and the primers were designed to amplify a fragment containing the predicted SREBF2 binding site. The primer sequences were:

Forward: 5'-GGTTGGTGCTCAATTTTCAGGA-3';

Reverse: 5'-GCTACCTAGCCCCTTCACTAC-3'.

### Co-immunoprecipitation (Co-IP)

Co-IP was performed using AML12 cells to assess protein interactions involving PPARα. Briefly, cells were lysed in ice-cold NP-40 lysis buffer (150 mM NaCl, 50 mM Tris-HCl, 1% NP-40, pH 7.8) supplemented with a mammalian protease inhibitor cocktail. Equal amounts of protein lysates (200 µg) were incubated overnight at 4 °C with anti-PPARα antibody (or normal mouse IgG as a negative control) on a nutating platform. Antibody-protein complexes were captured using Protein A/G agarose beads (Santa Cruz Biotechnology) for 1 hour at 4 °C with gentle agitation. Beads were washed four times with lysis buffer, and bound proteins were eluted by boiling in SDS loading buffer. Eluates were resolved by SDS-PAGE and analyzed by Western blot for O-GlcNAcylated proteins and OGT.

### RNA sequencing and data analysis

For *in vivo* RNA sequencing (RNA-seq), total RNA was extracted using TRIzol^®^ reagent (Invitrogen) from three control (Ctrl) and three 2% cholesterol (Chol) mouse liver samples. *In vitro* RNA-seq was also performed in AML12 cell experiments: untreated (UT, n=3) and MβCD-cholesterol-treated (4 μM, n=4) cells. Library preparation and sequencing were conducted on the Illumina NovaSeq 6000 platform by Shanghai Majorbio Bio-Pharm Technology Co., Ltd. Bioinformatic analyses, including differential expression and functional enrichment, were performed using the Majorbio I-Sanger Cloud Platform (https://www.i-sanger.com). (See **supplementary information** for details).

### Quantitative real-time polymerase chain reaction (qRT-PCR)

Total RNA was extracted using TRIzol^®^ reagent (Invitrogen, Carlsbad, CA) and reverse-transcribed into cDNA with kits from Promega (Madison, WI), following the manufacturer's instructions. qPCR was performed in 384-well plates using SYBR Green/ROX qPCR Master Mix (2X) on an Applied Biosystems 7300 Real-Time PCR System (Thermo Fisher Scientific). Gene expression levels were normalized to 18s rRNA and calculated using the 2^^-ΔΔCt^ method. Primers were synthesized by IDT, and their sequences are listed in **Table 1**.

### PPARα transcription factor activity

Nuclear extracts (Thermo Scientific,78833) from cells and tissues were prepared using a nuclear extraction kit according to the manufacturer's instructions. (See **supplementary information** for details).

### Metabolomics analysis

Targeted metabolomic analysis of central carbon metabolism was performed using ultra-performance liquid chromatography coupled with tandem mass spectrometry (UPLC-MS/MS; Thermo Fisher Scientific). Liver tissues were extracted using a methanol/water (80:20, v/v) solution containing internal standards, and metabolites were quantified by multiple reaction monitoring. Data acquisition and processing were carried out by Shanghai Majorbio Bio-Pharm Technology Co., Ltd. using in-house databases and the Majorbio cloud platform (https://cloud.majorbio.com). (Please refer to **the Supplementary information** for details.)

### Western blot analysis

Cells and liver tissues were lysed in RIPA buffer containing protease/phosphatase inhibitors (Beyotime, China). Equal amounts of protein (~30 µg) were separated by SDS-PAGE and transferred to membranes. Standard immunoblotting was performed with primary antibodies and IRDye-conjugated secondary antibodies (LI-COR), and signals were quantified using the Odyssey CLx system. For detection of N-linked glycosylation, membranes were probed with concanavalin A (ConA, Sigma-Aldrich) and visualized by chemiluminescence (ECL). β-actin and Lamin B1 served as loading controls. (Please refer to the **Supplementary information** for details on the sources and catalog numbers of all specific antibodies.)

### Statistical analysis

All statistical analyses were performed using GraphPad Prism (version 9.0; GraphPad Software, San Diego, CA). Unless otherwise specified, experiments were independently repeated at least three times (n ≥ 3). Data are expressed as mean ± standard deviation (SD). For comparisons between two groups, normality of data distribution was first evaluated using the *Shapiro-Wilk* test. Normally distributed data were analyzed by a two-tailed unpaired Student's *t*-test, whereas non-normally distributed data were assessed using the *Mann-Whitney U* test. For comparisons among more than two groups, one-way analysis of variance (ANOVA) followed by *Tukey's post hoc* test was applied. When two independent variables were involved, a two-way ANOVA with *Sidak's post hoc* analysis was used. For nonparametric data across multiple groups, the *Kruskal-Wallis* test was followed by Dunn's multiple comparison test. Sample sizes and *p*-values are represented by individual data points in bar-scatter plots. A *p*-value < 0.05 was considered statistically significant.

## Results

**Figure 1:**
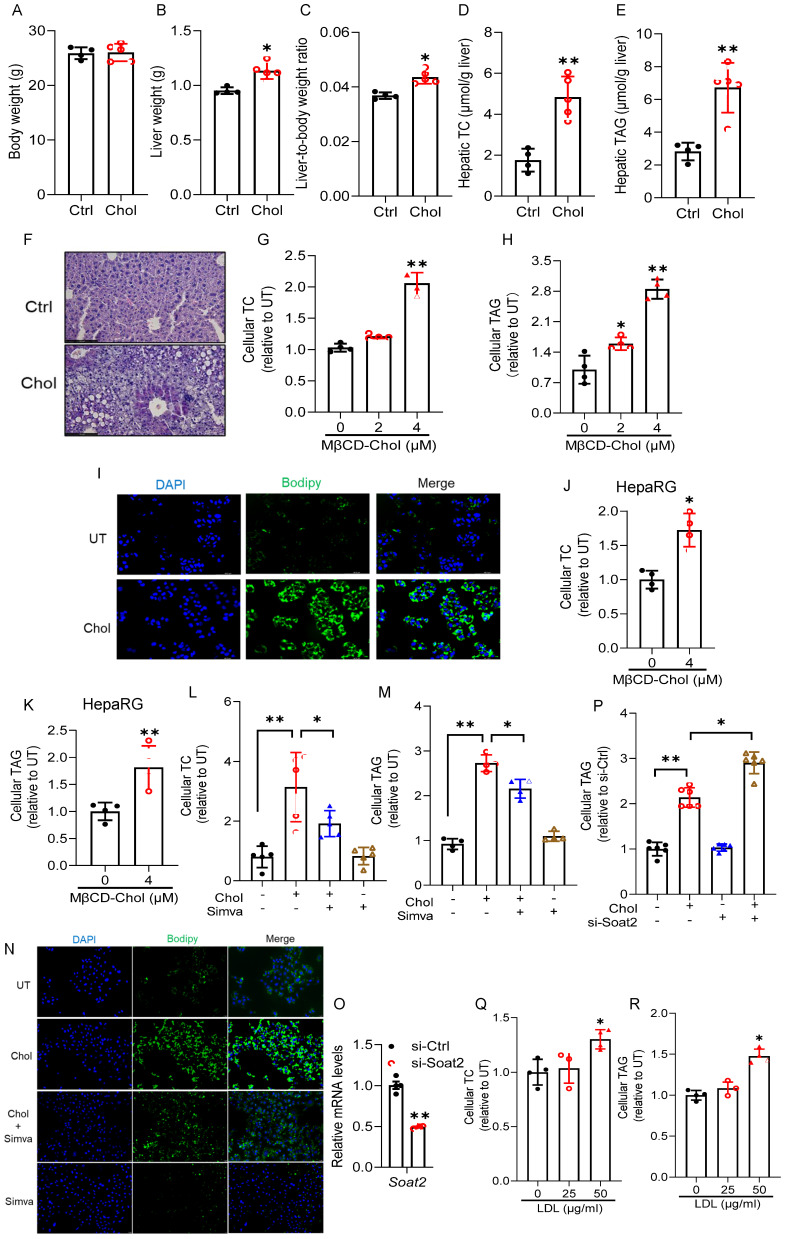
** Hepatocellular cholesterol overload promotes hepatic steatosis.** Male C57BL/6J mice (10-week-old) were fed with a chow diet supplemented with and without cholesterol (2%, w/w) for 4 weeks. (**A**) body weight; (**B**) liver weight; (**C**) liver-to-body weight ratio; (**D**) hepatic total cholesterol (TC) content; (**E**) hepatic triacylglycerol (TAG) content; (**F**) representative histology examination (H&E, Oil Red O, and Sirius Red staining). AML12 cells were treated with methyl-β-cyclodextrin (MβCD)-complexed cholesterol for 16 hours. (**G**) cellular total cholesterol (TC); (**H**) cellular TAG; (**I**) BODIPY staining (493/503), scale bar=50 μm. HepaRG cells were treated with methyl-β-cyclodextrin (MβCD)-complexed cholesterol for 16 hours. (**J**) cellular TC; (**K**) cellular TAG. AML12 cells were pretreated with simvastatin (10 μM) for 2 hours before MβCD-cholesterol loading. (**L**) cellular TC; (**M**) cellular TAG; (**N**) BODIPY staining (493/503). Scale bar=1 μm. AML12 cells were transfected with Soat2 siRNA overnight prior to MβCD-cholesterol loading. (**O**) *Soat2* mRNA; (**P**) cellular TAG. AML12 cells were treated with LDL (0-50 μg/mL) for 16 hours. (**Q**) cellular TC; (**R**) cellular TAG. Chol, cholesterol; MβCD-chol, MβCD-cholesterol; LDL, low-density lipoprotein; Simva, simvastatin. *P < 0.05 and **P < 0.01 represent statistical difference.

**Hepatocellular cholesterol overload promotes hepatic steatosis.** Both *in vivo* and *in vitro* studies were conducted to evaluate the effect of cholesterol overload on hepatic fat (triacylglycerol, TAG) accumulation. *In vivo*, 10-week-old male C57BL/6 mice were fed a standard chow diet with or without 2% (w/w) cholesterol for 4 weeks. As shown in **Fig. 1**, cholesterol supplementation did not alter body weight gain (**Fig. 1A**) but significantly increased liver mass (**Fig. 1B**), leading to a significant increase in the liver-to-body weight ratio (**Fig. 1C**)**.** As expected, hepatic total cholesterol (TC) content was markedly elevated (**Fig. 1D**). Importantly, dietary cholesterol supplementation induced hepatic steatosis, evidenced by increased hepatic TAG levels (**Fig. 1E**) and confirmed by histological staining (**Fig. 1F**).

To test whether cholesterol-induced hepatic TAG accumulation occurs through cell-autonomous mechanisms, we next examined the effects of cholesterol loading in cultured hepatocytes. AML12 cells, a non-transformed murine hepatocyte line, were first used, and key findings were validated in HepG2 and differentiated HepaRG cells to ensure translational relevance. In AML12 cells, treatment with methyl-β-cyclodextrin (MβCD)-complexed cholesterol significantly increased cellular TC (**Fig. 1G**) and TAG levels (**Fig. 1H**), as confirmed by BODIPY 493/503 staining (**Fig. 1I**). MβCD alone had no effect (**Figs. S1A, B**), ruling out vehicle artifacts. Similar results were observed in HepaRG (**Figs. 1J, K**) and HepG2 (**Figs. S1C-E**) cells, indicating a conserved, cell-autonomous effect of cholesterol loading. Moreover, pharmacological inhibition of endogenous cholesterol synthesis with simvastatin (10 μM) markedly reduced cholesterol-induced increases in cellular TC and TAG (**Figs. 1L-N**) in AML12 cells, with comparable effects in HepG2 cells (**Figs. S1F-H**). These findings confirm that elevated cellular cholesterol directly drives hepatocellular fat accumulation.

In hepatocytes, cholesterol exists in both free and esterified forms, with cholesteryl esters synthesized by acyl-CoA: cholesterol acyltransferase 2 (ACAT2, encoded by *Soat2*). To determine which pool contributes to TAG accumulation, *Soat2* was silenced in AML12 cells using siRNA (**Fig. 1O**). Remarkably, ACAT2 knockdown further enhanced MβCD-cholesterol-induced TAG accumulation (**Fig. 1P**), a result that was reproduced in HepG2 cells (**Fig. S1I**). These findings indicate that free cholesterol, rather than esterified cholesterol, is the major driver of lipid droplet formation, while ACAT2-mediated esterification serves a protective buffering role against free cholesterol-induced lipotoxicity. To assess physiological relevance, AML12 and HepG2 cells were treated with increasing concentrations of low-density lipoprotein (LDL, 0-50 μg/mL) for 16 hours. LDL supplementation dose-dependently increased cellular TC and TAG levels in both cell types (**Figs. 1Q**, **R, and Figs. S1J, K**), confirming that exogenous cholesterol promotes hepatocellular fat accumulation.

**Figure 2:**
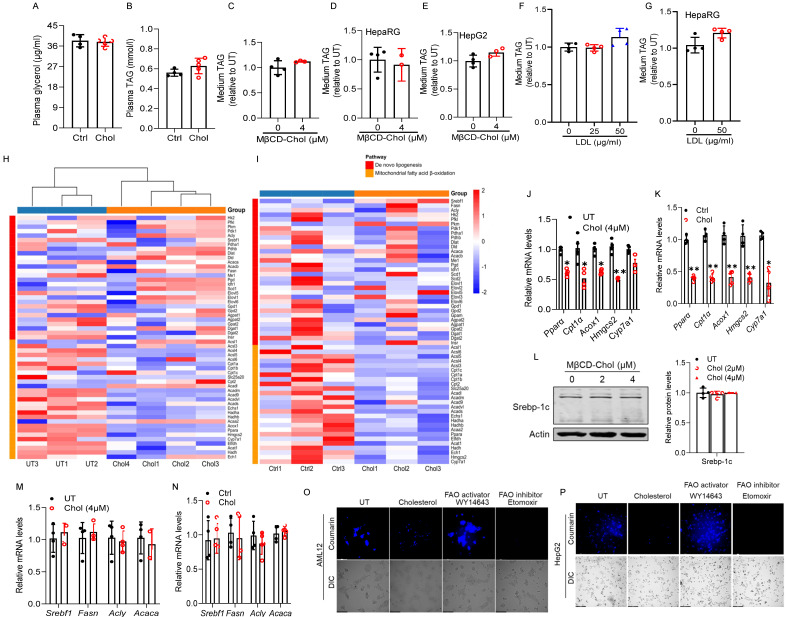
** Impaired mitochondrial fatty acid β-oxidation is a key metabolic defect contributing to cholesterol-induced hepatocellular fat accumulation.** (**A, B**) Plasma glycerol and TAG levels in chow-fed mice supplemented with and without 2% cholesterol (w/w) for 4 weeks. (**C-E**) Extracellular TAG concentrations in the culture medium of AML12, HepaRG, and HepG2 cells following cholesterol. (**F, G**) Extracellular TAG concentrations in the culture medium of AML12 and HepG2 cells following LDL supplementation. (**H, I**) Heatmap visualization of representative lipid-related pathways in AML12 cells and *in vivo*. (**J, K**) qRT-PCR validation of mitochondrial fatty acid β-oxidation (*Pparα*, *Cpt1α*, *Acox1*, *Hmgcs2*, and *Cyp7a1*) *in vitro* and *in vivo*. (**L-N**) Unaltered Srebp-1c pathway activation in AML12 cells following cholesterol exposure and in chow-fed mice supplemented with and without 2% cholesterol for 4 weeks, tested by Western-blot and qRT-PCR. (**O, P**) Fluorescence-based FAOBlue™ assay of fatty acid β-oxidation capacity (FAO) in AML12 and HepG2 cells after cholesterol loading (4 μM, 16 h). (40× objective). Chol, cholesterol; MβCD-chol, MβCD-cholesterol; LDL, low-density lipoprotein. *P < 0.05 and **P < 0.01 represent statistical significance.

**Impaired fatty acid β-oxidation is a key metabolic defect contributing to cholesterol-induced hepatocellular fat accumulation.** Excessive hepatic fat accumulation may result from multiple metabolic disturbances, including enhanced *de novo* lipogenesis (DNL), impaired mitochondrial β-oxidation, increased fatty acid influx from adipose tissue, or defective very low-density lipoprotein (VLDL) secretion 41, 42. In mice fed a cholesterol-enriched diet, plasma glycerol levels remained unchanged (**Fig. 2A**), excluding enhanced adipose lipolysis as a contributing factor. Moreover, a modest elevation in plasma TAG levels was observed (**Fig. 2B**), suggesting that hepatic TAG accumulation was not attributable to impaired VLDL exports. Consistently, cholesterol loading, via either MβCD-cholesterol or LDL exposure, did not affect extracellular TAG levels in AML12, HepaRG, or HepG2 cells (**Figs. 2C-G**), further indicating that VLDL secretion was preserved. To define how cholesterol promotes hepatic lipid accumulation, we conducted RNA-seq in cholesterol-treated AML12 cells (4 μM) and in the livers of mice fed a 2% (w/w) cholesterol diet for 4 weeks. Transcriptomic analysis showed that cholesterol did not alter the expression of DNL-related genes but markedly suppressed mitochondrial fatty acid β-oxidation genes in both models (**Figs. 2H, I**). These findings were confirmed by qRT-PCR in both settings (**Figs. 2J, K**). The lack of changes in mature SREBP-1c protein levels *in vitro* and *in vivo* after cholesterol loading (**Figs. 2L-N**) further supports that cholesterol-induced lipid accumulation occurs independently of DNL activation. In contrast, consistent with the transcriptomic downregulation of fatty acid β-oxidation genes, fluorescence-based assessment of fatty acid oxidation using the FAOBlue^TM^ assay demonstrated that cholesterol exposure markedly impaired mitochondrial β-oxidation in both AML12 and HepG2 cells (**Figs. 2O, P**). Collectively, these findings establish that defective mitochondrial β-oxidation, rather than enhanced lipogenesis or impaired VLDL export, is the principal mechanism driving cholesterol-induced hepatic TAG accumulation.

**Figure 3:**
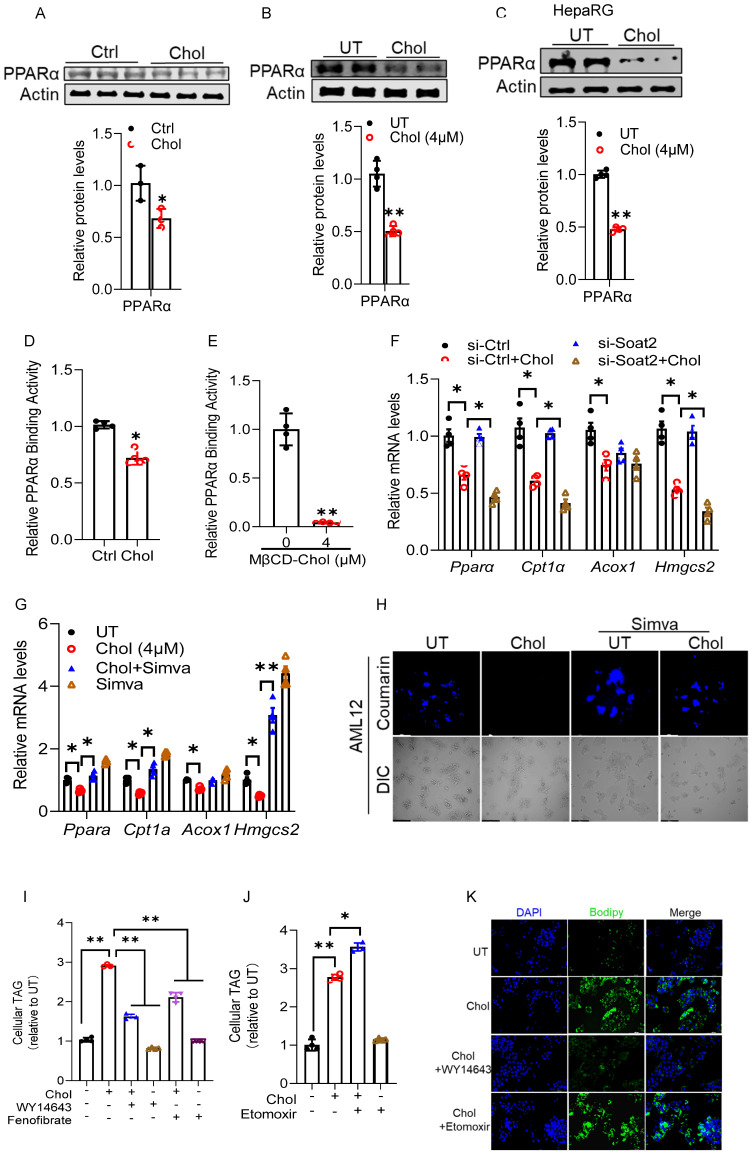
** Hepatocellular free cholesterol overload suppresses PPARα signaling, contributing to intracellular lipid accumulation.** Cholesterol-fed mice and MβCD-cholesterol-treated hepatocytes were examined for PPARα signaling. (**A-C**) Western blot analysis of PPARα protein levels in livers of cholesterol-fed mice and in AML12 and HepaRG cells exposed to cholesterol. (**D, E**) ELISA-based measurement of hepatic and cellular PPARα transcriptional activity. (**F**) qRT-PCR analysis of PPARα target genes in AML12 cells following siRNA-mediated *Soat2* knockdown prior to cholesterol loading. (**G**) Effects of simvastatin (10 μM) pretreatment on PPARα protein expression and target gene induction in cholesterol-treated AML12 cells. (**H**) FAOBlue^TM^ assay showing restoration of mitochondrial fatty acid β-oxidation by simvastatin in AML12 cells. (**I**) Intracellular TAG in AML12 cells treated with WY14643 or fenofibrate during cholesterol loading. (**J**) TAG accumulation in AML12 cells treated with the FAO inhibitor etomoxir. (**K**) BODIPY staining showing reduced lipid droplets with WY14643 and increased droplets with etomoxir. Scale bar=50 μm. Chol, cholesterol; MβCD-Chol, MβCD-cholesterol; Simva, simvastatin. *P < 0.05 and **P < 0.01 represent statistical significance*.*

**Hepatocellular free cholesterol overload suppresses PPARα signaling, contributing to intracellular lipid accumulation.** One of the key regulators of hepatic fatty acid β-oxidation is PPARα. Our RNA-sequencing analysis revealed that its expression is downregulated in the liver following cholesterol-enriched diet feeding (**Figs. 2H-K**). We therefore next examined whether cholesterol impairs hepatic PPARα signaling. Western blot analysis revealed a pronounced reduction in PPARα protein abundance in the livers of mice fed a cholesterol-enriched diet (**Fig. 3A**) and in cultured hepatocytes treated with cholesterol (**Figs. 3B, C,** and **Fig. S2A**). Consistently, hepatic PPARα transcriptional activity, measured using an ELISA-based assay, was significantly diminished in both cholesterol-fed mice (**Fig. 3D**) and cholesterol-treated AML12 cells (**Fig. 3E**). Similar inhibitory effects were also observed in AML12 cells treated with LDL (**Figs. S2B, C**). Notably, *Soat2* knockdown further exacerbated cholesterol-induced repression of PPARα target genes (**Fig. 3F**), implicating the accumulation of free cholesterol as a key driver of PPARα inhibition. Conversely, simvastatin (10 μM) pretreatment restored PPARα expression and activation of its target genes (**Fig. 3G**), as well as rescued fatty acid β-oxidation inhibition, determined by FAOBlue staining, in cultured hepatocytes (**Fig. 3H** and **Fig. S2D**). To determine whether PPARα activation mechanistically contributes to cholesterol-induced hepatocellular fat accumulation, we conducted rescue experiments using selective PPARα agonists in cultured hepatocytes. In cholesterol-loaded AML12 cells, activation of PPARα with WY14643 (10 μM) or fenofibrate (20 μM) markedly attenuated intracellular fat accumulation (**Fig. 3I**). In contrast, inhibition of mitochondrial fatty acid β-oxidation with etomoxir (20 μM) further exacerbated TAG accumulation under the same conditions (**Fig. 3J**). As a result, BODIPY staining revealed fewer lipid droplets in WY14643-treated cells, whereas etomoxir treatment intensified lipid deposition (**Fig. 3K**).

**Figure 4:**
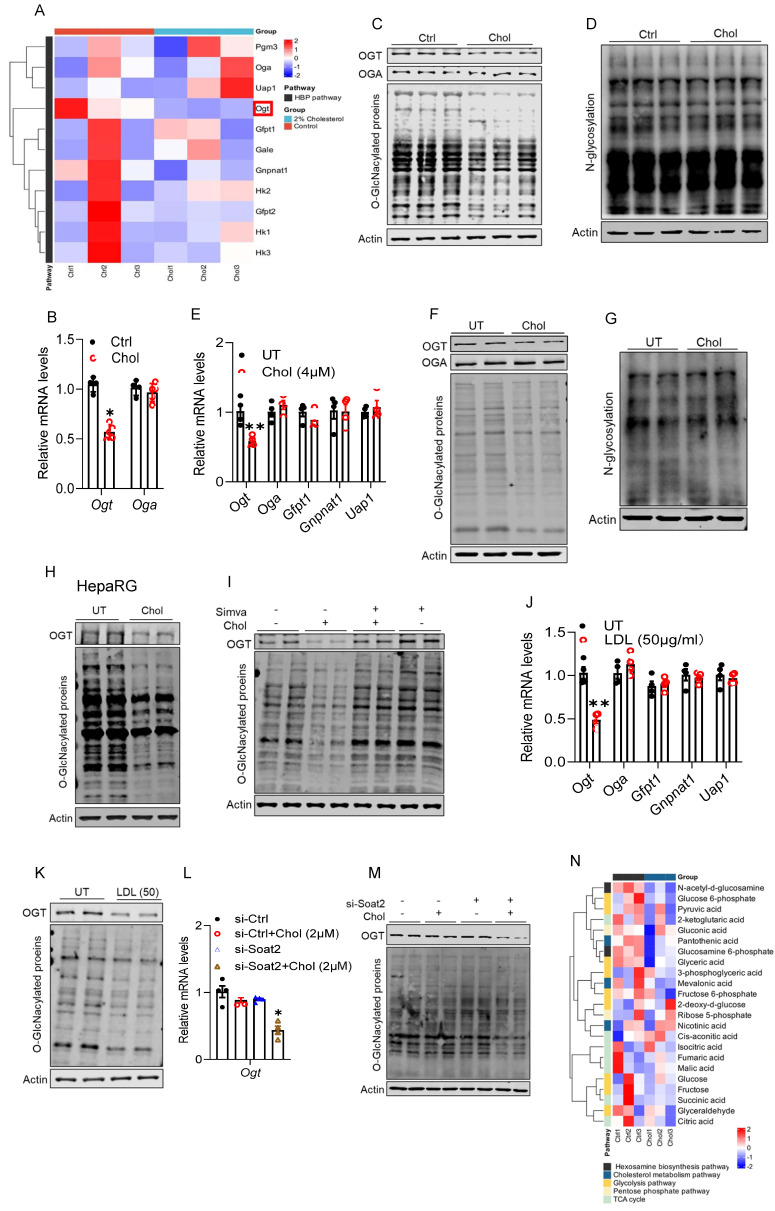
** Cholesterol loading downregulates hepatic OGT expression and suppresses hepatic protein O-GlcNAcylation.** Male C57BL/6J mice (10-week-old) were fed with a chow diet supplemented with and without cholesterol (2%, w/w) for 4 weeks. (**A**) Heatmap of transcriptomic profiling. (**B, C**) Validation by qRT-PCR and Western blot. (**D**) Lectin blot analysis showing unchanged N-linked glycosylation in the same samples. AML12 cells were treated with methyl-β-cyclodextrin (MβCD)-complexed cholesterol for 16 hours. (**E-G**) Validation by qRT-PCR and Western blot. (**H**) A similar result was observed in HepaRG cells. (**I**) Simvastatin (10 μM, pretreatment for 2 h) reversed the cholesterol-induced reduction of OGT and global O-GlcNAcylation in AML12 cells. (**J, K**) LDL supplementation (50 μg/mL, 16 h) decreased OGT mRNA and protein levels and suppressed O-GlcNAcylation in AML12 cells. (**L, M**) siRNA-mediated knockdown of *Soat2* further suppressed OGT expression and exacerbated the cholesterol (MβCD-chol, 2 μM, 16 h)-induced decrease in global O-GlcNAcylation in AML12 cells. (**N**) Targeted metabolomic analysis of liver tissues. Chol, cholesterol; O-GlcNAc, O-GlcNAcylation. *P < 0.05 and **P < 0.01 represent statistical significance*.*

**Cholesterol loading downregulates hepatic OGT expression and suppresses hepatic protein O-GlcNAcylation.** We previously demonstrated that activation of the hexosamine biosynthetic pathway (HBP) is essential for glucose-induced hepatic TAG accumulation 43, underscoring its central role in hepatic lipid metabolism. Transcriptomic profiling of liver tissues from cholesterol-fed mice revealed a selective downregulation of *Ogt* (**Fig. 4A**), which encodes O-linked N-acetylglucosamine transferase (OGT), the sole enzyme catalyzing protein O-GlcNAcylation using the HBP-derived substrate UDP-GlcNAc. This suppression was highly specific, as expression of other core HBP enzymes (*Gfpt1, Gnpnat1, Uap1*) and *Oga* (encoding O-GlcNAcase) remained unchanged (**Fig. 4A**). Subsequent qRT-PCR and Western blot analyses confirmed these transcriptomic findings. In cholesterol-fed mouse livers, OGT expression was significantly reduced at both the mRNA (**Fig. 4B**) and protein levels, accompanied by a marked decrease in global protein O-GlcNAcylation (**Fig. 4C and Fig. S3A**), while N-linked glycosylation remained unchanged (**Fig. 4D and Fig. S3B**). Similarly, in AML12 hepatocytes, cholesterol loading with MβCD-cholesterol (4 μM) markedly suppressed OGT gene expression (**Fig. 4E**), without affecting other HBP enzymes. This was associated with a parallel reduction in global protein O-GlcNAcylation (**Fig. 4F and Fig. S3C**) but no change in N-linked glycosylation (**Fig. 4G and Fig. S3D**). Consistent results were observed in HepaRG and HepG2 cells (**Fig. 4H and Figs. S3E, F**). Notably, pharmacological depletion of intracellular cholesterol with simvastatin restored OGT expression and normalized protein O-GlcNAcylation in cultured hepatocytes (**Fig. 4I and Figs. S3G, H**). Comparable rescue effects were observed when LDL served as the cholesterol source (**Figs. 4J, K, and Fig. S3I**). Moreover, *Soat2* knockdown, which increases free cholesterol levels, further reduced OGT expression and exacerbated O-GlcNAcylation loss (**Figs. 4L, M, and Fig. S3J**), reinforcing free cholesterol as the primary mediator of this effect. Consistent with these molecular findings, targeted metabolomics revealed that dietary cholesterol supplementation significantly reduced hepatic levels of glucosamine-6-phosphate and N-acetyl-D-glucosamine, two key intermediates in the HBP (**Fig. 4N**).

**Figure 5:**
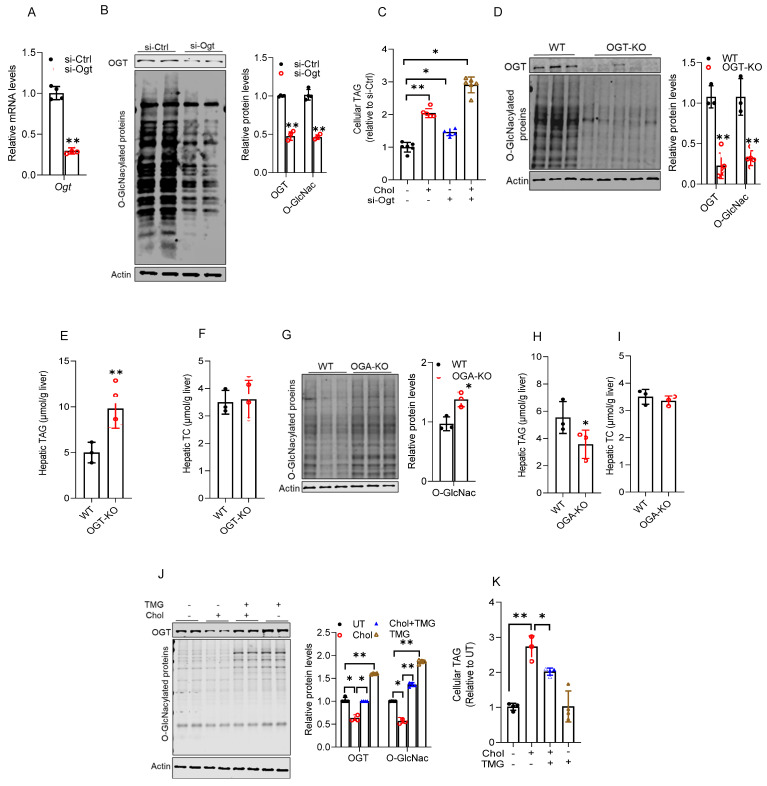
** Impairment of O-GlcNAcylation underlies cholesterol-induced hepatic fat accumulation.** AML12 cells were transfected with siRNA targeting *Ogt* overnight and then treated with methyl-β-cyclodextrin-cholesterol (4 μM, 16 h). (**A**) hepatic *Ogt* mRNA. (**B**) global protein O-GlcNAcylation and OGT protein; (**C**) cellular TAG content. Liver-specific *Ogt* or *Oga* knockout mice were used to examine *in vivo* effects. (**D**) hepatic global protein O-GlcNAcylation and OGT protein; (**E**) hepatic TAG; (**F**) hepatic TC; (**G**) hepatic global protein O-GlcNAcylation; (**H**) hepatic TAG; (**I**) hepatic TC. AML12 cells were pretreated with TMG for 2 hours prior to cholesterol loading. (**J**) global protein O-GlcNAcylation and OGT protein; (**K**) cellular TAG. Chol, cholesterol; O-GlcNAc, O-GlcNAcylation. *P < 0.05 and **P < 0.01 represent statistical significance*.*

**Impairment of O-GlcNAcylation underlies cholesterol-induced hepatic fat accumulation.** To determine whether cholesterol-induced OGT downregulation and subsequent suppression of protein O-GlcNAcylation contribute to hepatic lipid accumulation, we first inhibited OGT activity in hepatocytes using siRNA-mediated knockdown. OGT downregulation markedly reduced global protein O-GlcNAcylation, which was accompanied by a significant increase in cellular TAG content in both AML12 (**Figs. 5A-C**) and HepG2 cells (**Figs. S4A, B**). Consistently, hepatocyte-specific OGT knockout mice exhibited inhibited hepatic protein O-GlcNAcylation (**Fig. 5D**), concomitant with pronounced TAG accumulation, without changes in hepatic TC levels (**Figs. 5E, F**). In contrast, hepatocyte-specific OGA knockout mice, in which protein O-GlcNAcylation is elevated (**Fig. 5G**), manifested reduced TAG levels while maintaining normal hepatic TC content (**Figs. 5H, I**). Together, these findings demonstrate that OGT-mediated protein O-GlcNAcylation plays a critical role in maintaining hepatic lipid homeostasis. To further test this mechanism, we pharmacologically enhanced O-GlcNAcylation prior to cholesterol loading by pretreating AML12 and HepG2 cells with Thiamet-G (TMG), an OGA inhibitor that prevents O-GlcNAc removal from proteins. In line with prior observations, cholesterol exposure markedly decreased global protein O-GlcNAcylation in both cell types, and this effect was effectively reversed by TMG pretreatment (**Fig. 5J and Fig. S4C**). Importantly, restoration of O-GlcNAcylation significantly attenuated cholesterol-induced TAG accumulation (**Fig. 5K and Fig. S4D**).

**Figure 6:**
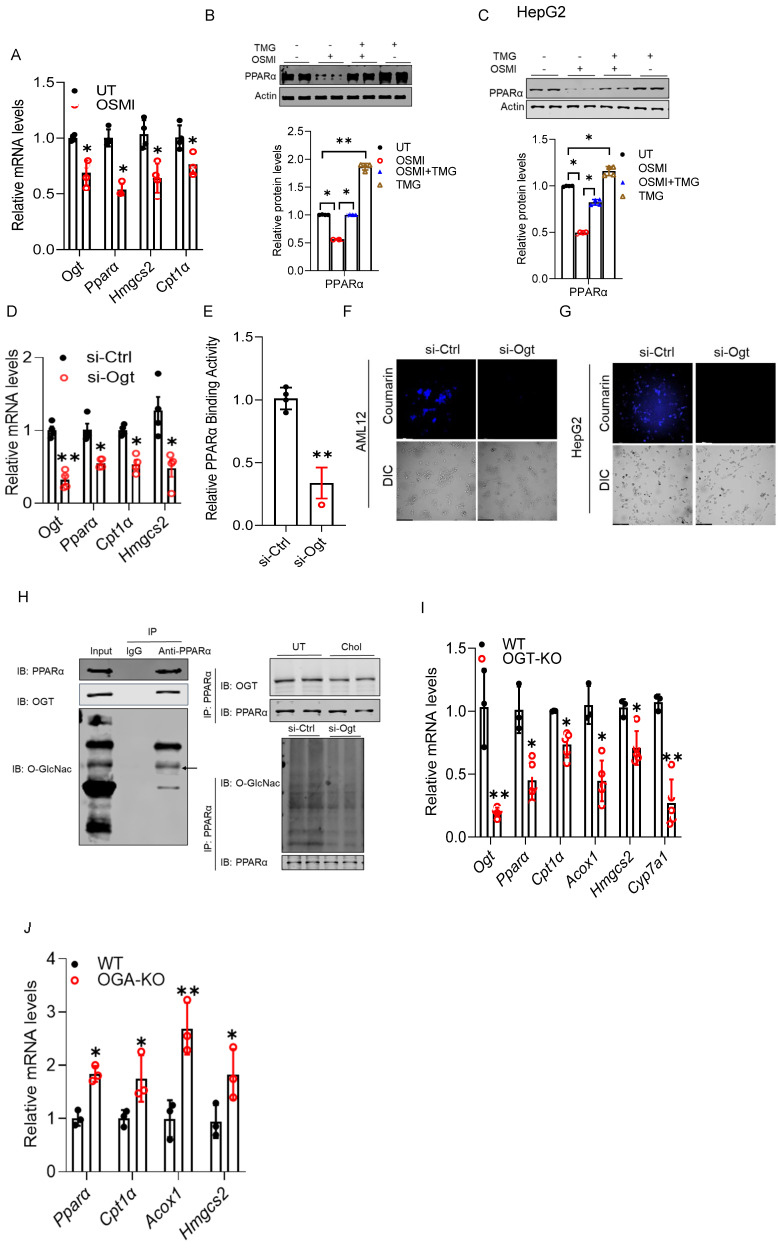
** OGT-mediated O-GlcNAcylation is essential for hepatic PPARα transactivation.** AML12 cells were treated with OSMI-1. (**A**) qRT-PCR analysis of *Ogt*, *Pparα*, *Hmgcs2*, and *Cpt1a* mRNA levels. AML12 and HepG2 cells were treated with OSMI-1, either alone or in combination with TMG. (**B, C**) Western blot analyses of PPARα protein. (**D, E**) qRT-PCR and ELISA analyses showing that *Ogt* silencing reduced *Pparα*, *Hmgcs2*, and *Cpt1α* expression and decreased PPARα transcriptional activity in AML12 cells. (**F, G**) FAOBlue^TM^ assay in AML12 and HepG2 cells. PPARα O-GlcNAcylation was examined by co-immunoprecipitation. (**H**) OGT association with PPARα (IB: OGT) and O-GlcNAc modification of PPARα (IB: O-GlcNAc; arrow). Right: effects of cholesterol treatment or si*Ogt* on PPARα O-GlcNAcylation. (**I, J**) qRT-PCR analysis of hepatic *Pparα*, *Hmgcs2*, *Cpt1α*, *Acox1*, and *Cyp7a1* mRNA levels in liver-specific OGT- and OGA-deficient mice. O-GlcNAc, O-GlcNAcylation. *P < 0.05 and **P < 0.01 represent statistical significance.

**OGT-mediated O-GlcNAcylation is essential for hepatic PPARα transactivation.** Our data show that hepatocellular cholesterol overload inhibits both PPARα transactivation and OGT-mediated protein O-GlcNAcylation, and that these impairments contribute to hepatic lipid accumulation. These findings led us to hypothesize that PPARα transactivity is regulated by protein O-GlcNAcylation. To explore this potential link, we manipulated OGT and OGA expression and activity using both pharmacological and genetic approaches in cultured hepatocytes and subsequently assessed PPARα transactivity. As shown in **Figs. 6A-C**, the OGT inhibitor (OSMI) reduced PPARα expression at both the mRNA and protein levels, accompanied by a coordinated downregulation of canonical PPARα target genes, including those involved in mitochondrial β-oxidation. Importantly, co-treatment with the OGA inhibitor, TMG (10 µM), restored OSMI-suppressed PPARα expression (**Fig. 6B**). Similar findings were observed in HepG2 cells (**Fig. 6C**). Consistently, genetic inhibition of *Ogt* via siRNA transfection recapitulated the effects of pharmacological inhibition on *Pparα* expression and its canonical downstream targets (**Fig. 6D**). This observation was further supported by a direct ELISA-based transactivity assay (**Fig. 6E**), as well as reduced fatty acid β-oxidation, as evidenced by FAOBlue staining in cultured hepatocytes (**Figs. 6F, G**). Co-immunoprecipitation (CO-IP) revealed that PPARα physically interacts with OGT and is directly O-GlcNAcylated (**Fig. 6H**), indicating a potential post-translational regulatory mechanism. Lastly, to establish *in vivo* relevance, we assessed hepatic PPARα activity in liver-specific OGT-deficient mice. In line with our cell-based findings, hepatic *Ogt* deficiency reduced* Pparα* expression and its canonical downstream targets (**Fig. 6I**). In contrast, *Oga* knockout in mice led to an opposite transcriptional pattern, as evidenced by increased expression of *Pparα* and its downstream targets (**Fig. 6J**). Collectively, these data identify OGT-mediated protein O-GlcNAcylation as a key mechanism required to sustain PPARα transactivation.

**Figure 7:**
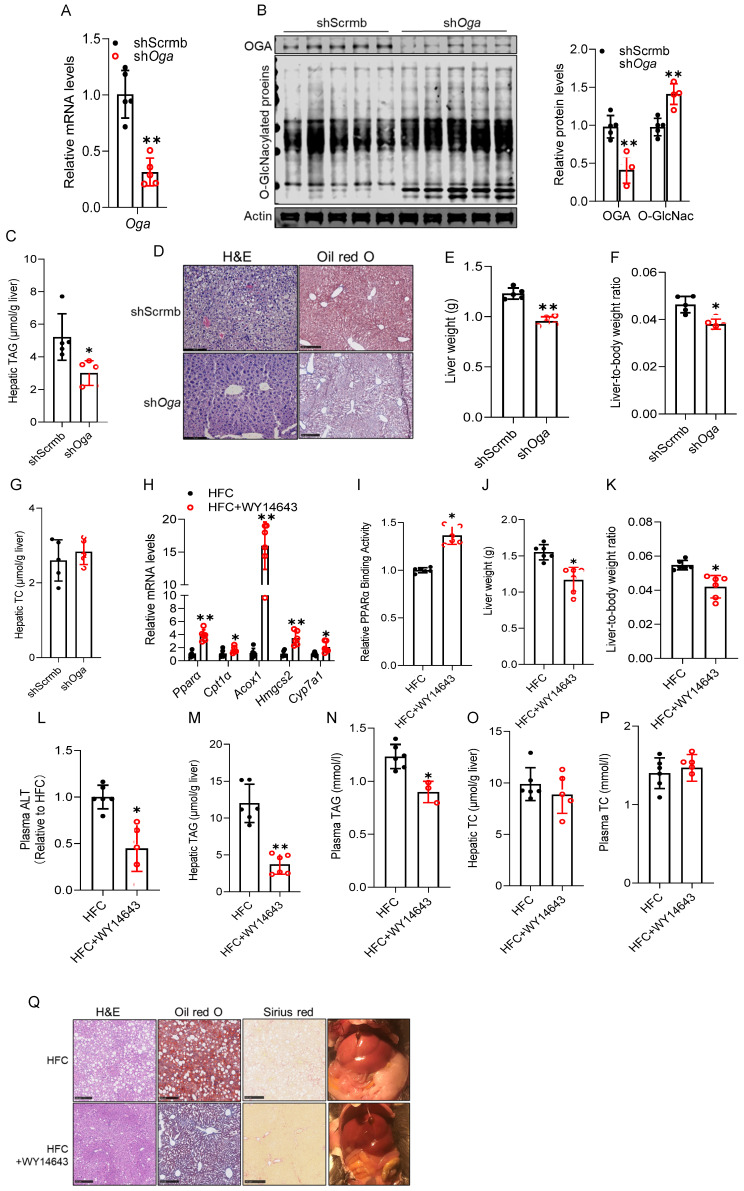
**Either the improvement of protein O-GlcNAcylation or PPARα activation attenuates cholesterol-induced hepatic steatosis.** C57BL/6 male mice (10-week-old) were fed a 2% cholesterol diet for 30 days. On day 10, mice received a single retro-orbital injection of AAV8-shScramble or AAV8-sh*Oga*. (**A**) hepatic *Oga* mRNA; (**B**) hepatic OGA protein and global protein O-GlcNAcylation; (**C**) hepatic TAG; (**D**) representative liver images, H&E and Oil Red O staining; (**E**) liver weight; (**F**) liver-to-body weight ratio; (**G**) hepatic TC. C57BL/6 mice fed a high-fat diet supplemented with 2% cholesterol (HFC) and treated with WY14643. (**H**) hepatic mRNA expression of canonical *Pparα* target genes; (**I**) Nuclear PPARα transactivation; (**J**) liver weight; (**K**) liver-to-body weight ratio; (**L**) Plasma alanine aminotransferase (ALT) levels; (**M, N**) hepatic and plasma TAG; (**O, P**) hepatic and plasma TC; (**Q**) representative liver morphology and histological analyses (H&E, Oil Red O, and Sirius Red staining. Chol, cholesterol; O-GlcNAc, O-GlcNAcylation. Chol, cholesterol; MβCD-Chol, MβCD-cholesterol; Simva, simvastatin. *P < 0.05 and **P < 0.01 represent statistical significance*.*

**Either the improvement of protein O-GlcNAcylation or PPARα activation attenuates cholesterol-induced hepatic steatosis.** To evaluate the translational relevance of our mechanistic findings, we investigated whether cholesterol diet-induced hepatic steatosis can be ameliorated by enhancing protein O-GlcNAcylation or by pharmacological activation of PPARα in mice. To this end, 10-week-old male C57BL/6 mice were fed a 2% cholesterol-supplemented diet for 30 days and received a single retro-orbital injection of either AAV8-shScramble or AAV8-shOga on day 10 to increase hepatic O-GlcNAcylation via OGA knockdown. AAV8-shOga delivery effectively reduced hepatic OGA expression and ameliorated cholesterol-induced inhibition of protein O-GlcNAcylation (**Figs. 7A, B**), which was associated with marked improvements in cholesterol-induced hepatic abnormalities, including decreased fat accumulation (**Figs. 7C, D**), reduced liver weight (**Fig. 7E**), and a lower liver-to-body weight ratio (**Fig. 7F**), without affecting hepatic total cholesterol levels (**Fig. 7G**). To assess whether pharmacological activation of PPARα could rescue MASLD progression, male C57BL/6 mice were fed a high-fat diet (HF; 35% kcal from fat) supplemented with 2% (w/w) cholesterol (HFC) for 6 weeks. WY14643 (30 mg/kg/day) was administered beginning in week 4 and continued throughout the final three weeks of dietary challenge. WY14643 treatment robustly activated hepatic PPARα signaling, as evidenced by upregulation of canonical PPARα target genes (**Fig. 7H**) and enhanced nuclear PPARα transactivation (**Fig. 7I**). Restoration of PPARα function was associated with a marked improvement in the hepatic phenotype, including reduced liver weight and the liver-to-body weight ratio (**Figs. 7J, K**), as well as decreased plasma ALT levels (**Fig. 7L**), indicating attenuated hepatocellular injury. Both hepatic (**Fig. 7M**) and circulating TAG concentrations (**Fig. 7N**) were significantly reduced, while total cholesterol levels in the liver and plasma remained unchanged (**Figs. 7O, P**), suggesting that the therapeutic benefits of PPARα activation were independent of systemic cholesterol burden.

Histological analyses, including H&E, Oil Red O, and Sirius Red staining, further confirmed the protective role of PPARα activation (**Fig. 7Q**). Consistently, gross examination also confirmed improved liver appearance in WY14643-treated mice.

**Figure 8:**
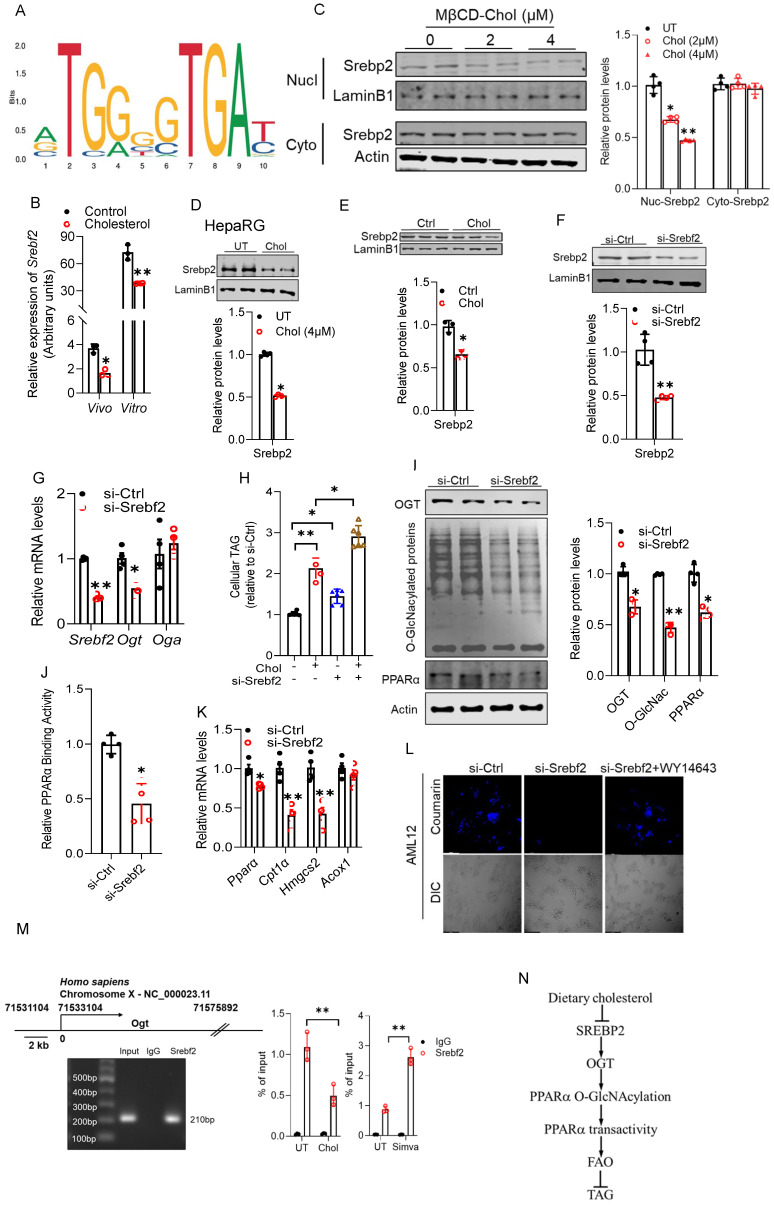
** SREBP2 regulates OGT expression and mediates cholesterol-induced suppression of protein O-GlcNAcylation and PPARα signaling.** Integrative transcriptomic analysis was performed to identify transcriptional regulators of *Ogt*. (**A, B**) RNA-seq pathway analysis combined with JASPAR motif prediction identified SREBP-2 as a candidate regulator of *Ogt*. SREBP-2 expression in hepatocytes and mouse liver. (**C-E**) Western blot analysis of SREBP-2 protein in AML12 cells, HepaRG cells, and livers of cholesterol-fed mice. Functional analysis of *Srebf2* knockdown in AML12 cells treated with or without MβCD-cholesterol. (**F**) Western blot of SREBP-2 protein; (**G**) qRT-PCR of *Srebf2*, *Ogt*, and *Oga*; (**H**) intracellular TAG content; (**I**) Western blot and quantification of global O-GlcNAcylation, OGT, and PPARα; (**J**) ELISA-based PPARα transcriptional activity; (**K**) qRT-PCR of canonical PPARα target genes. (**L**) FAOBlue^TM^ assay showing FAO activity following* Srebf2* knockdown, with or without co-treatment with the PPARα agonist WY14643. (**M**) ChIP-qPCR analysis demonstrates direct binding of SREBP-2 to the *Ogt* promoter (~2 kb upstream of the transcription start site). (**N**) Schematic summary of the proposed mechanism. Chol, cholesterol; O-GlcNAc, O-GlcNAcylation. *P < 0.05 and **P < 0.01 represent statistical significance*.*

**SREBP2 regulates OGT expression and mediates cholesterol-induced suppression of protein O-GlcNAcylation and PPARα signaling.** To elucidate the molecular mechanism underlying cholesterol-induced OGT suppression, we performed an integrative analysis combining RNA-seq-derived transcriptomic data with JASPAR transcription factor binding predictions. This analysis identified sterol regulatory element-binding protein 2 (SREBP2) as a putative transcriptional regulator of *Ogt* (**Fig. 8A**). Consistent with RNA-seq data (**Fig. 8B**), cholesterol loading via either MβCD-cholesterol or LDL treatment consistently inhibited SREBP2 activation across all hepatocyte models used in this study and liver tissues (**Figs. 8C-E and Figs. S5A, B**). To assess the functional relevance of SREBP2, we silenced *Srebf2* in AML12 and HepG2 cells using siRNA (**Figs. 8F, G, and Fig. S5C**). *Srebf2* knockdown increased intracellular fat accumulation under basal conditions, which was further exacerbated by cholesterol loading (4 μM) (**Fig. 8H and Fig. S5D**). This phenotype was accompanied by reduced OGT expression, decreased global protein O-GlcNAcylation, and impaired PPARα signaling, as reflected by reduced PPARα protein abundance and transcriptional activity (**Figs. 8I, J, and Fig. S5E**), along with downregulation of canonical PPARα target genes (**Fig. 8K**). Functionally, *Srebf2* silencing markedly decreased fatty acid oxidation (FAO) capacity, an effect that was partially rescued by WY14643, a selective PPARα agonist (**Fig. 8L and Fig. S5F**). To determine whether SREBP2 directly regulates *Ogt* transcription, we performed chromatin immunoprecipitation (ChIP) using primers targeting the -2 kb region upstream of the *Ogt* transcription start site, where SREBP2 binding motifs were predicted. ChIP-qPCR analysis confirmed direct binding of SREBP2 to the *Ogt* promoter (**Fig. 8M**). Collectively, these findings identify SREBP2 as a direct transcriptional activator of OGT and demonstrate that cholesterol suppresses hepatic OGT expression by inhibiting SREBP2 activity. This repression impairs O-GlcNAc-dependent mitochondrial fatty acid β-oxidation, at least in part by inhibiting PPARα signaling.

## Discussion

Although dietary cholesterol has long been implicated in MASLD progression 8, 44, whether and how cholesterol loading drives hepatocellular fat accumulation has remained unclear. Here, we identify a previously unrecognized mechanism by which free cholesterol promotes hepatic steatosis by suppressing PPARα-dependent fatty acid β-oxidation. Mechanistically, hepatocellular cholesterol overload downregulates OGT expression, leading to reduced global protein O-GlcNAcylation, including that of PPARα. This loss of O-GlcNAcylation impairs PPARα transactivity, thereby compromising mitochondrial fatty acid β-oxidation and promoting intracellular lipid accumulation. Importantly, restoration of protein O-GlcNAcylation via hepatic OGA knockdown or pharmacological activation of PPARα effectively attenuated cholesterol-induced steatosis in both cultured hepatocytes and mouse models (**Fig. 8N**). Collectively, these findings establish a cholesterol-OGT-O-GlcNAc-PPARα axis linking cholesterol overload to impaired fatty acid oxidation and hepatic steatosis and highlight O-GlcNAc signaling and PPARα as promising therapeutic targets.

Although dietary cholesterol is well recognized as a major driver of advanced MASLD, contributing to inflammation, liver injury, and fibrosis, collectively termed cholesterol-associated steatohepatitis (CASH) 8, 45-47, its role in promoting hepatocellular fat accumulation and hepatic steatosis, the earliest stage of MASLD, has remained poorly defined. Our findings address this gap using both *in vivo* and *in vitro* models. In cell culture studies, we demonstrate that exogenous cholesterol, supplied either as free cholesterol (MβCD-cholesterol) or in its physiologically relevant LDL-bound form, is sufficient to induce hepatocellular fat accumulation independent of changes in *de novo* lipogenesis (DNL), adipose tissue lipolysis, or VLDL secretion. The specificity of this mechanism is supported by two key observations: (1) simvastatin treatment, which reduces intracellular cholesterol, markedly attenuates cellular fat accumulation under our experimental treatments; and (2) silencing of *Soat2*, which blocks cholesterol esterification and expands the free cholesterol pool, further exacerbates TAG accumulation. These effects were consistently observed across multiple hepatic models, including murine AML12, human HepG2, and HepaRG cells, and were validated in our animal studies, in which mice were fed cholesterol-supplemented chow or high-fat diets. Importantly, hepatic TAG accumulation increased in a dose-dependent manner with respect to cholesterol, without concurrent changes in body weight or systemic metabolic parameters, indicating a direct, liver-specific effect of cholesterol overload.

Mechanistically, our study identifies mitochondrial fatty acid oxidation inhibition as a central driver of cholesterol-induced hepatocellular fat accumulation. To delineate the underlying mechanism, we focused on PPARα, a key transcriptional regulator governing mitochondrial and peroxisomal β-oxidation. Our findings demonstrate that hepatocellular free cholesterol overload markedly suppresses PPARα transactivation. This conclusion is supported by converging evidence, including reduced PPARα expression, downregulation of canonical PPARα-targeting genes, and direct measurements of diminished transcriptional activity. These data indicate that cholesterol accumulation disrupts PPARα-dependent metabolic programming, thereby impairing fatty acid catabolism. Importantly, pharmacological activation of PPARα using structurally distinct agonists effectively restored β-oxidation capacity and prevented lipid accumulation in cholesterol-overloaded hepatocytes. These results not only establish PPARα inhibition as a key mechanistic link between cholesterol overload and defective fatty acid oxidation but also highlight PPARα as a potential therapeutic target for mitigating cholesterol-driven hepatic steatosis.

Another interesting finding of the present study is that cholesterol overload suppresses OGT-mediated protein O-GlcNAcylation. To our knowledge, this is the first study to establish a direct association between hepatic cholesterol overload and OGT downregulation. O-GlcNAcylation is a reversible, nutrient-sensitive post-translational modification catalyzed by OGT and removed by OGA 29, 48. Although O-GlcNAc signaling has been primarily studied in the context of glucose-responsive pathways such as SREBP-1c- and mTORC1-mediated lipogenesis 49, 50, its role in cholesterol-induced metabolic alterations has remained largely unexplored. Here, we demonstrate that intracellular cholesterol accumulation suppresses OGT expression at both the mRNA and protein levels, leading to global reductions in protein O-GlcNAcylation. Notably, these cholesterol-induced defects are functionally reversible; either pharmacological inhibition of OGA or genetic deletion of hepatic *Oga* restores global protein O-GlcNAcylation and alleviates cholesterol-induced steatosis. Conversely, hepatocyte-specific *Ogt* deletion phenocopies the metabolic effects of a cholesterol-enriched diet. Collectively, these findings define O-GlcNAc signaling as a critical regulatory axis linking cholesterol overload to hepatic steatosis.

The observation that hepatocellular free cholesterol overload is associated with both PPARα inhibition and suppression of OGT-mediated protein O-GlcNAcylation prompted us to further explore the mechanistic connection between O-GlcNAcylation and PPARα transactivation. Our results identify PPARα as a key downstream target whose activity is regulated by O-GlcNAcylation. PPARα is a master transcriptional regulator of mitochondrial and peroxisomal β-oxidation pathways, and its activity is modulated by ligand binding, phosphorylation, and other post-translational modifications 10, 11, 16. Our results demonstrate that O-GlcNAcylation positively regulates PPARα transcriptional activity, providing an additional layer of control over hepatic lipid catabolism. Co-immunoprecipitation (Co-IP) confirmed that PPARα is directly O-GlcNAcylated, and loss of this modification resulted in reduced DNA-binding affinity and diminished transcriptional activation of β-oxidation genes. Restoring protein O-GlcNAcylation reinstated PPARα activity and fatty acid β-oxidation gene expression, linking O-GlcNAcylation status to hepatic fatty acid β-oxidation capacity.

Integrative transcriptomic and ChIP analyses identified SREBP2 as a direct transcriptional activator of *Ogt*, demonstrating that SREBP2 binds the *Ogt* promoter to regulate its expression. Both cholesterol loading in cultured hepatocytes and dietary cholesterol supplementation in mice suppressed hepatic SREBP2 activity, consistent with the canonical feedback regulation of cholesterol homeostasis 51, 52. This repression provides a mechanistic explanation for the downregulation of OGT in cholesterol-overloaded livers, extending the functional repertoire of SREBP2 beyond cholesterol biosynthesis and positioning it upstream of a cholesterol-OGT-fatty acid β-oxidation axis that governs hepatic metabolic flexibility. This pathway defines a regulatory checkpoint through which cholesterol impairs mitochondrial fatty acid β-oxidation and promotes steatosis, independent of canonical lipogenic mechanisms. While our data establishes that exogenous or dietary cholesterol disrupts O-GlcNAcylation via SREBP2 inhibition, hepatic cholesterol can also accumulate through non-dietary mechanisms, including increased endogenous synthesis, impaired biliary excretion, or enhanced cholesteryl ester hydrolysis. A recent study further demonstrated that caspase-2-mediated SREBP2 activation, together with feedback circuits involving LATS2, sustains hepatic cholesterol accumulation 53. Whether these alternative routes converge on the same SREBP2-OGT axis remains to be determined.

In summary, our study delineates a previously unrecognized mechanism underlying cholesterol-induced hepatocellular fat accumulation and hepatic steatosis. Cholesterol-mediated downregulation of OGT and consequent loss of protein O-GlcNAcylation impair PPARα transactivation, leading to the inhibition of mitochondrial fatty acid β-oxidation and subsequent hepatic fat accumulation. Importantly, restoring protein O-GlcNAcylation or pharmacologically activating PPARα effectively reverses these metabolic defects, demonstrating the causal role of this pathway in the development of fatty liver. Unlike FFAs, which have long been recognized as the primary drivers of hepatic steatosis due to their direct contribution to TAG synthesis and lipotoxic effects, cholesterol is not a direct substrate for TAG synthesis. The present study provides evidence that, by inhibiting mitochondrial fatty acid oxidation, cholesterol can act in concert with FFAs, both elevated in MASLD, to promote hepatic lipid accumulation and synergistically contribute to the development and progression of MASLD. Collectively, our findings establish O-GlcNAc signaling as a central metabolic checkpoint linking cholesterol sensing to lipid oxidation and suggest that targeting OGT-mediated O-GlcNAcylation or PPARα activation may represent promising therapeutic strategies for cholesterol-driven fatty liver disease.

## Supplementary Material

Supplementary materials and methods, figures.

## Figures and Tables

**Table 1:** The sequences of all primers.

Target genes	Forward primer (5′to 3′)	Reverse primer (5′to 3′)
Mouse *Oga*	GGGTTATGGAGCAGAGAAAAGAG	CCTGGCGAAATAGCATAGATGAA
Mouse *Ogt*	GACGCAACCAAACTTTGCAGT	TCAAGGGTGACAGCCTTTTCA
Mouse *Soat2*	ACAAGACAGACCTCTTCCCTC	ATGGTTCGGAAATGTTGCACC
Mouse *Fasn*	GGAGGTGGTGATAGCCGGTAT	TGGGTAATCCATAGAGCCCAG
Mouse *Acaca*	GATGAACCATCTCCGTTGGC	GACCCAATTATGAATCGGGAGTG
Mouse *Acly*	CAGCCAAGGCAATTTCAGAGC	CTCGACGTTTGATTAACTGGTCT
Mouse *srebf1*	GCAGCCACCATCTAGCCTG	CAGCAGTGAGTCTGCCTTGAT
Mouse *srebf2*	CAGGTGCAGACGGTACAGG	CGACCCTTACTGGCACTTGAA
Mouse *Pparα*	AGAGCCCCATCTGTCCTCTC	ACTGGTAGTCTGCAAAACCAAA
Mouse *Hmgcs2*	GAAGAGAGCGATGCAGGAAAC	GTCCACATATTGGGCTGGAAA
Mouse *Cpt1α*	CTCCGCCTGAGCCATGAAG	CACCAGTGATGATGCCATTCT
Mouse *Acox1*	TCCAGACTTCCAACATGAGGA	CTGGGCGTAGGTGCCAATTA
Mouse *Cyp7a1*	GGGATTGCTGTGGTAGTGAGC	GGTATGGAATCAACCCGTTGTC
Mouse* Gfpt1*	GAAGCCAACGCCTGCAAAATC	CCAACGGGTATGAGCTATTCC
Mouse *Gnpnat1*	ATGAAACCCGATGAAACTCCC	GCCTCAAAACCAAGCCTTCTC
Mouse *Uap1*	CCATCCCCCGCTTGAAAGAT	CCCTCTCCAGCATAAGAGATGA
Mouse *18s*	TAACCCGTTGAACCCCATT	CCATCCAATCGGTAGTAGCG

## Data Availability

All data and materials are available.

## Abbreviations

FFAs: free fatty acids
MASLD: metabolic dysfunction-associated steatotic liver disease
OGT: O-linked N-acetylglucosamine transferase
OGA: O-GlcNAcase
PPARα: peroxisome proliferator-activated receptor alpha
SREBP2: sterol regulatory element-binding protein-2
TAG: triacylglycerol
TC: total cholesterol
TMG: thiamet G
HBP: hexosamine biosynthetic pathway
FAO: fatty acid oxidation
ALT: alanine aminotransferase
ACAT2: alanine aminotransferase
