# Supplementary material for: Cholesterol Overload Drives Hepatic Steatosis by Inhibiting OGT-dependent PPARα O-GlcNAcylation and Transactivation
Source: Int J Biol Sci. 2026 May 18;22(11):5615–33. doi: 10.7150/ijbs.135054 (PMC13282704; doi:10.7150/ijbs.135054)

Cholesterol Overload Drives Hepatic Steatosis by Inhibiting OGT-dependent PPARα O-GlcNAcylation and Transactivation

Rui Guo[1,2,*], Yanhui Li[1,*], Qinchao Ding[2], Chad Slawson[3], Udayan Apte[4], Yuwei Jiang[5], Xiaobing Dou[6], Songtao Li[2], Zhenyuan Song[1]

[1]Department of Kinesiology and Nutrition, University of Illinois Chicago, Chicago, IL, USA.

[2]School of Public Health, Zhejiang Chinese Medical University, Hangzhou, Zhejiang, PR. China.

[3]Department of Biochemistry and Molecular Biology, Kansas University Medical Center, Kansas City, KS, USA

[4]Department of Pharmacology, Toxicology & Therapeutics, Kansas University Medical Center, Kansas City, KS, USA

[5]Department of Physiology & Biophysics, University of Illinois Chicago, Chicago, IL, USA

[6]School of Life Science, Zhejiang Chinese Medical University, Hangzhou, Zhejiang, PR. China.

Supplementary materials and methods……………..2

Supplementary figure legends………………………...6

Supplementary materials and methods

Cell culture studies:

Murine AML12 and human HepG2 hepatocyte cell lines were obtained from the American Type Culture Collection (ATCC, VA, USA). AML12 cells were cultured in DMEM/F12 medium (Gibco, Waltham, MA) supplemented with 10 % fetal bovine serum (FBS, J. R. Scientific, Woodland, CA), 100 U/mL penicillin, 0.1 mg/mL streptomycin, 40 ng/mL dexamethasone, and an ITS supplement (5 mg/L insulin, 5 mg/L transferrin, and 5 µg/L selenium). HepG2 cells were maintained in DMEM with 10% FBS. Human hepatoma HepaRG cells were rapidly thawed in a 37 °C water bath, transferred into 10 mL of pre-warmed William's E Medium (no phenol red, Thermo Fisher Scientific) supplemented with the HepaRG Supplement Pack (Thermo Fisher Scientific), 1 mM sodium pyruvate, and 2 mM L-glutamine. The cell suspension was centrifuged at 1200 rpm for 5 min at room temperature to remove DMSO. After centrifugation, the pellet was resuspended in complete HepaRG culture medium. Prior to cell seeding, sterile 24-well plates pre-coated with type I collagen were rinsed gently three times with sterile phosphate-buffered saline (PBS, without calcium and magnesium). Cells were then seeded at a density of 1.2 × 10^5 cells per well in 500 µL of complete medium. All cell lines were grown in 75 cm² flasks at 37 °C in a humidified incubator with 5% CO_2 and treated at ~ 70% confluence for subsequent experiments.

RNA interference:

Transfection was performed according to Invitrogen's Lipofectamine 2000 protocol. In brief, cells were cultured to 75% confluence in a 6-well culture plate and transfected with 5 µL siRNA (10 mM) using 8 µL Lipofectamine 2000 or 24-well plate transfected with 1.25 µL siRNA (10 mM) using 2 µL Lipofectamine 2000. As a control, cells were transfected with scrambled RNA under the same conditions. The transfection result was assessed by real-time quantitative PCR (qRT-PCR) after 16 hours of siRNA transfection.

Fatty acid oxidation (FAO) staining:

Cells were seeded in 12-well plates and cultured under experimental conditions. After treatment, the cultured medium was removed, and cells were washed twice with HEPES-buffered saline (HBS). Cells were then incubated with 5 µM FAOBlue™ working solution (1:500 dilution in HBS) at 37 °C for 60 min in the dark. Following staining, cells were washed twice with warm HBS to remove excess dye. Blue fluorescence (Ex. 405 nm/ Em. 430-480 nm) was detected using a

fluorescence microscope. Images were acquired and analyzed using identical settings across all groups.

Animal experiments:

Cohort 1: Ten-week-old male C57BL/6 mice (n=9). Four mice were fed standard chow (control), and five mice were fed chow supplemented with 2% cholesterol for 4 weeks.

Cohort 2: Ten-week-old male C57BL/6 mice. Groups: High-fat diet (HFD)+2% cholesterol (n=5) and HFD+2% cholesterol plus WY14643 (Cayman, 70730, 30 mg/kg/day in drinking water, n=5) for 6 weeks.

Cohort 3: Hepatocyte-specific OGT knockout (OGT-KO) and OGA knockout (OGA-KO) mice were generated by breeding $OGT^{flox/flox}$ and $OGA^{flox/flox}$ mice (kindly provided by Dr. Chad Slawson from the University of Kansas Medical Center) with albumin-Cre mice (Jackson Laboratory, Bar Harbor, ME). The methodology for creating OGT-KO and OGA-KO mice has been previously described in (Boyd, Robarts *et al.*, 2024). Experimental cohorts comprised three littermate controls and five OGT-KO mice, and three littermate controls and three OGA-KO mice, respectively.

Cohort 4: Recombinant adeno-associated virus serotype 8 (AAV8) vectors carrying a U6 promoter-driven short hairpin RNA (shRNA) targeting murine Oga (AAV8-sh*Oga*) or a scrambled control shRNA (AAV8-shScrmb) were constructed by VectorBuilder (Chicago, IL, USA). The shRNA sequence targeting *Oga* (5'-ATAGTGTCTGCAGGATTATTA-3') was cloned into a pAAV backbone (pAAV[shRNA]-CAG>EGFP-U6>mOga[shRNA#1]), in which EGFP was included as a reporter. The viral particles were packaged into AAV serotype 8 capsids and purified, yielding a genomic titer >1×10^13 GC/mL. Ten-week-old male C57BL/6 mice received a single retro-orbital injection of AAV8-shScrmb (n=5) or AAV8-sh*Oga* (n=5) (100 µL per mouse) on day 10 of dietary intervention. Mice were then maintained on a 2% cholesterol diet for 30 days before sacrifice.

All mice were euthanized by carbon dioxide (CO_2) inhalation followed by cervical dislocation, in accordance with institutional guidelines and approved animal care protocols. Liver tissues were rapidly excised; nuclear and cytoplasmic proteins were isolated using the NE-PER™ Nuclear and Cytoplasmic Extraction Reagents (Thermo Scientific, 78833) according to the manufacturer's protocol. Additional liver samples were snap-frozen in liquid nitrogen and stored at -80 °C for

subsequent analyses. Blood was collected from the inferior vena cava, centrifuged at 3,000 × g for 15 min at 4 °C, and the resulting plasma was aliquoted and stored at -80 °C until use.

Biochemical assays:

For liver samples, approximately 100 mg of tissue was homogenized in 500 μL of 50 mM NaCl. Lipids were extracted using a hexane/isopropanol mixture (3:2, v/v). After adding the solvent, samples were gently shaken on a platform shaker at room temperature for 2 hours in the dark. The upper organic phase was then collected and air-dried under a fume hood. TAG and TC levels were quantified using commercial reagents (Jiancheng, Nanjing, China) according to the manufacturer's instructions.

Plasma TAG and TC were measured using commercially available TAG and Cholesterol Reagent Set (Jiancheng, Nanjing, China). Plasma glycerol concentrations were measured using a Glycerol Colorimetric Assay Kit (Cayman, Ann Arbor, MI). Plasma ALT levels were determined by an ALT determination kit (Biobyt, orb1173221, NC, USA).

For cellular lipid quantification, cells were seeded into 24-well plates at a density of 1-2 × 10^5 cells per well and lysed with 300 μL of 0.25 M NaOH. Lipid extraction and quantification were performed using the same protocol as for liver tissues. Protein concentrations in cell lysates were measured using a BCA protein assay kit (Thermo Scientific, USA), and TAG/TC levels were normalized to total protein content. For TAG and TC detection in the culture medium (supernatant), protein normalization was not required, as equal volumes of culture medium were used across all groups.

RNA sequencing and data analysis:

Total RNA was isolated from mouse liver tissues (three biological replicates per group: Control and 2% Cholesterol) and AML12 cells (cholesterol treatment: UT, n=3; 4 μM cholesterol, n=4) using TRIzol® reagent (Invitrogen, Carlsbad, CA, USA) according to the manufacturer's protocol. RNA concentration and purity were measured using a NanoDrop 2000 spectrophotometer (Thermo Fisher Scientific, Waltham, MA, USA), and RNA integrity was verified by an Agilent 2100 Bioanalyzer (Agilent Technologies, Santa Clara, CA, USA). RNA-seq libraries were prepared and sequenced on an Illumina NovaSeq 6000 platform (paired-end, 150 bp) by Shanghai Majorbio Bio-Pharm Technology Co., Ltd. (Shanghai, China). Clean reads were aligned to the mouse reference genome (GRCm39) using HISAT2, and transcript abundance was quantified as fragments per kilobase of exon model per million mapped reads (FPKM) using StringTie.

Differentially expressed genes were identified based on $|\log_2(\text{fold change})| \geq 1$ and adjusted P value < 0.05. All analyses were performed on the Majorbio I-Sanger Cloud Platform (https://www.i-sanger.com).

PPARα transcription factor activity:

PPARα DNA-binding activity was assessed using the PPARα transcription factor assay kit (Cayman, 10006915) following the provided protocol. Briefly, nuclear extracts were added to a 96-well plate pre-coated with a PPARα-specific DNA response element. After incubation and washing, a primary antibody specific to PPARα was applied, followed by an HRP-conjugated secondary antibody. The bound transcription factor was detected using a colorimetric substrate, and the absorbance was measured at 450 nm using a microplate reader. The results were normalized to the total nuclear protein input in both *in vivo* and *in vitro* conditions.

Metabolomics analysis:

For targeted metabolomic analysis of central carbon metabolism, liver tissues (~30 mg) were homogenized in ice-cold methanol/water (80:20, v/v) containing internal standards. After centrifugation at 15,000 × g for 15 min at 4 °C, the supernatants were collected and analyzed by liquid chromatography-tandem mass spectrometry. Chromatographic separation was performed on a C18 reverse-phase column with a binary solvent system consisting of water (0.1% formic acid) and acetonitrile/isopropanol (1:1, v/v, 0.1% formic acid) under gradient elution. Mass spectrometry detection was performed on a UPLC-Q Exactive system (Thermo Fisher Scientific) operating in both positive and negative ionization modes using multiple reaction monitoring (MRM). Data acquisition and peak integration were processed using Progenesis QI software (Waters Corporation, Milford, USA). Multivariate statistical analyses, including principal component analysis (PCA) and orthogonal partial least squares discriminant analysis (OPLS-DA), were performed with the R package ropls (version 1.6.2). Differential metabolites were identified based on variable importance in projection (VIP > 1) and Student's *t*-test ($P < 0.05$).

Western blot analysis:

For protein extraction, cultured cells were lysed in RIPA buffer (Beyotime, China) supplemented with protease and phosphatase inhibitors, whereas liver tissues were homogenized in enhanced RIPA buffer (Beyotime, China) containing the same inhibitors. After incubation on ice for 30 min, lysates were centrifuged at 12,000 × g for 10 min at 4 °C to remove debris, and protein concentrations were determined using a BCA assay kit.

Equal amounts of protein (~30 μg) were separated by 10% SDS-PAGE and transferred to nitrocellulose membranes (LI-COR, USA). For immunoblotting, membranes were blocked and incubated overnight at 4 °C with primary antibodies, including OGT (1:1000, CST, 24083S), O-GlcNAc (1:1000, CST, 82332S), MGEA5/OGA (1:1000, Invitrogen, PA5-67426), Srebp1 (1:1000, ABclonal, A15586), Srebp2 (1:1000, ABclonal, A25685), PPARα (1:1000, Santa Cruz, sc-398394), Lamin B1 (1:1000, ABclonal, A1910), and β-actin (1:1000, CST, 4970L). After washing, the membranes were incubated with IRDye-conjugated secondary antibodies (LI-COR) for 1 hour at room temperature. Protein bands were visualized using the Odyssey CLx imaging system and quantified with ImageStudio software. To detect N-linked glycosylation, membranes after transfer were briefly blocked with 0.5% Tween-20 in PBS for 5 min at room temperature and then incubated overnight at 4 °C with ConA (Sigma-Aldrich, 5 μg/mL). Following PBS washes, N-linked glycosylation signals were visualized with enhanced chemiluminescence reagents (Thermo Fisher Scientific) using a Tanon chemiluminescence imaging system. β-actin and Lamin B1 served as loading controls for cytoplasmic and nuclear proteins, respectively. Target protein levels were normalized to the corresponding internal controls and expressed relative to the untreated group, which was set to be 1. All experiments were performed in triplicate for statistical reliability.

Supplementary figure legends

Figure S1. Hepatocellular cholesterol overload promotes hepatic steatosis. (**A, B**) Cellular TC and TAG levels in AML12 cells treated with MβCD-cholesterol or vehicle MβCD alone. HepG2 cells were treated with methyl-β-cyclodextrin (MβCD)-complexed cholesterol for 16 hours. (**C**) cellular total cholesterol (TC); (**D**) cellular triacylglycerol (TAG); (**E**) BODIPY staining (493/503), scale bar=50 μm. HepG2 cells were pretreated with simvastatin (10 μM) for 2 hours before MβCD-cholesterol loading. (**F**) cellular TC; (**G**) cellular TAG; (**H**) BODIPY staining (493/503), scale bar=1 μm. (**I**) *Soat2* knockdown enhances cholesterol-induced TAG accumulation in HepG2 cells. (**J, K**) LDL supplementation increases cellular TC and TAG in HepG2 cells. Chol, cholesterol; MβCD-chol, MβCD-cholesterol; LDL, low-density lipoprotein; Simva, simvastatin. *$P < 0.05$ and **$P < 0.01$ represent statistical difference.

Figure S2. Hepatocellular free cholesterol overload suppresses PPARα signaling, contributing to intracellular lipid accumulation. (**A**) Quantification of PPARα protein expression associated with cholesterol treatment in HepG2 cells. (**B**) The protein abundance of PPARα in AML12 cells following LDL treatment. (**C**) The mRNA expression of *Ppara* and its target genes in AML12 cells following LDL treatment. (**D**) Effect of simvastatin (10 μM) pretreatment on Fluorescence-based FAOBlueTM assay of fatty acid β-oxidation capacity (FAO) in HepG2 cells after cholesterol loading (4 μM, 16 h). (40× objective). **P* < 0.05 and ***P* < 0.01 represent statistical difference.

Figure S3. Cholesterol loading downregulates hepatic OGT expression and suppresses hepatic protein O-GlcNAcylation. (**A-D**) Quantification of protein expression and glycosylation changes associated with cholesterol treatment *in vivo* and *in vitro*. Densitometric quantification of immunoblots in Figure 4C and D showing hepatic protein OGT and O-GlcNAcylation (**A**), as well as N-linked glycosylation (**B**) levels in control and cholesterol-fed mice. Densitometric quantification of immunoblots in Figure 4F-H showing OGT, global protein O-GlcNAcylation (**C**), and N-linked glycosylation (**D**) levels in AML12 cells treated with cholesterol (4 μM) complexed with MβCD. (**E, F**) Validation of cholesterol-induced OGT suppression in human hepatocyte models. HepaRG and HepG2 cells exposed to MβCD-cholesterol (MβCD-chol, 4 μM, 16 h) showed decreased OGT protein abundance with concomitant reduction of global protein O-GlcNAcylation. (**G, H**) Simvastatin (10 μM, pretreatment for 2 h) reversed the cholesterol-induced reduction of OGT and global O-GlcNAcylation in AML12 and HepG2 cells. (**I**) Densitometric quantification of immunoblots in Figure 4K showing OGT and global protein O-GlcNAcylation in AML12 cells treated with LDL. (**J**) Densitometric quantification of immunoblots in Figure 4M showing OGT expression and global protein O-GlcNAcylation levels in AML12 cells treated with MβCD-cholesterol (2 μM, 16 h) in the presence or absence of siRNA-mediated *Soat2* knockdown. Simva, simvastatin; Chol, cholesterol; O-GlcNAc, O-GlcNAcylation. **P* < 0.05 and ***P* < 0.01 represent statistical significance.

Figure S4. Impairment of O-GlcNAcylation underlies cholesterol-induced hepatic fat accumulation. (**A, B**) siRNA-mediated depletion of OGT reduced global protein O-GlcNAcylation, which collectively exacerbated cholesterol (4 μM, 16 h)-induced intracellular TAG accumulation in HepG2 cells. (**C**) Representative Western blots and quantification showing

global protein O-GlcNAcylation and OGT protein levels. (**D**) Cellular TAG content following cholesterol exposure with or without TMG pretreatment in HepG2 cells. Chol, cholesterol; O-GlcNAc, O-GlcNAcylation. *$P < 0.05$ and **$P < 0.01$ represent statistical significance.

Figure S5. SREBP2 regulates OGT expression and mediates cholesterol-induced suppression of protein O-GlcNAcylation and PPARα signaling. (**A**) Western blot and quantification of Srebp2 protein levels in cholesterol-induced HepG2 cells. (**B**) The protein abundance of Srebp2 in AML12 cells following LDL treatment. HepG2 cells were transfected with siRNA targeting *Srebf2* or control siRNA and treated with or without 4 μM cholesterol (16 h). (**C, D**) Western blot and quantification of Srebp2 protein levels, and intracellular TAG content were measured. (**E**) Western blot and quantification of global protein O-GlcNAcylation, OGT, and PPARα protein levels. (**F**) FAO capacity measured in HepG2 cells, with or without co-treatment with the PPARα agonist WY14643, following *Srebf2* knockdown. Chol, cholesterol; LDL, low-density lipoprotein; O-GlcNAc, O-GlcNAcylation. *$P < 0.05$ and **$P < 0.01$ represent statistical significance.

Reference:

Boyd SS, Robarts DR, Nguyen K, Villar M, Alghusen IM, Kotulkar M, Denson A, Fedosyuk H, Whelan SA, Lee NCY, Hanover J, Dias WB, Tan EP, McGreal SR, Artigues A, Swerdlow RH, Thompson JA, Apte U and Slawson C. Multi-omics after O-GlcNAc alteration identified cellular processes promoting aneuploidy after loss of O-GlcNAc transferase. Mol Metab 2024;90:102060

Suppl. Figure 1

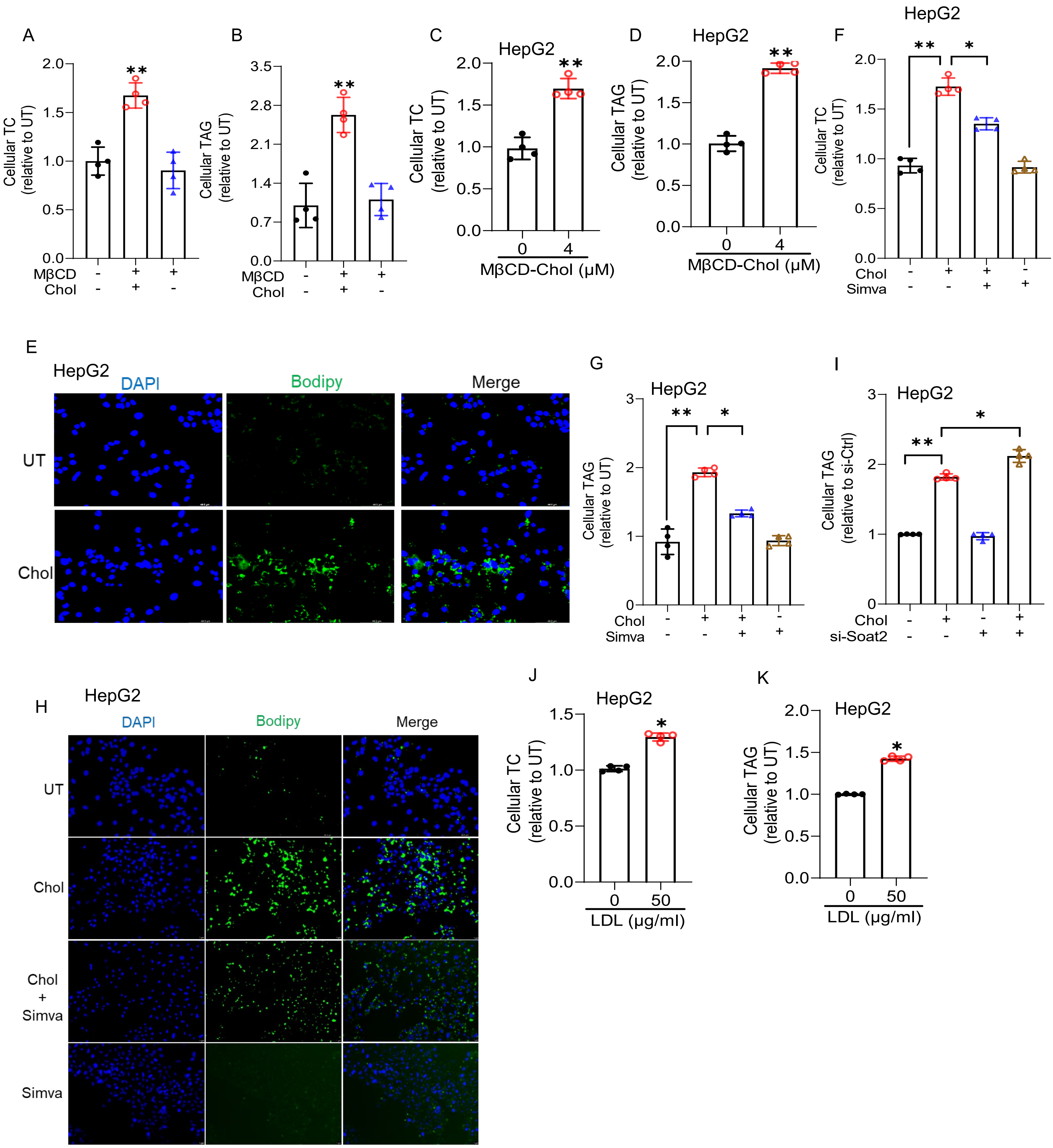

Suppl. Figure 2

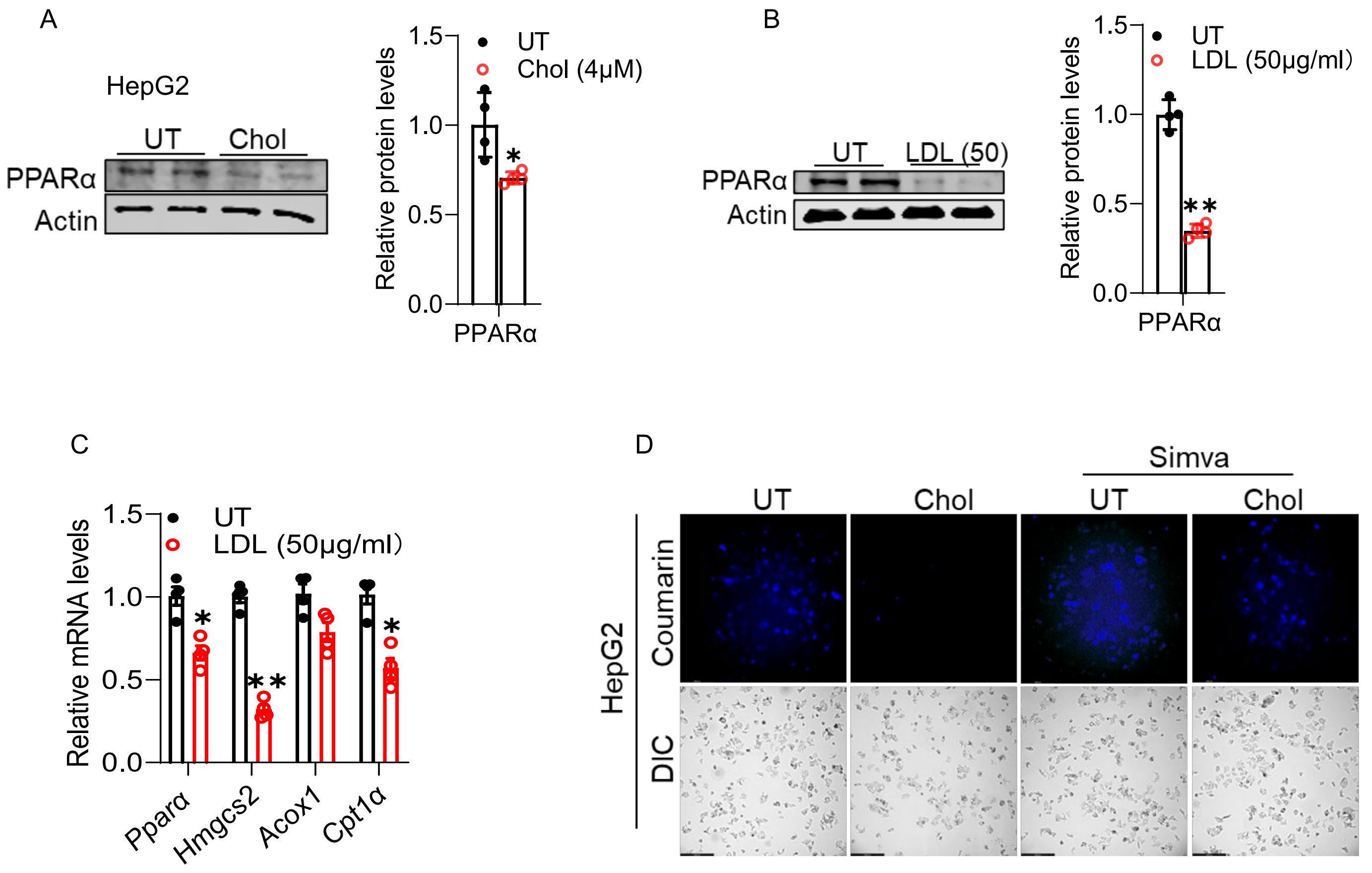

Suppl. Figure 3

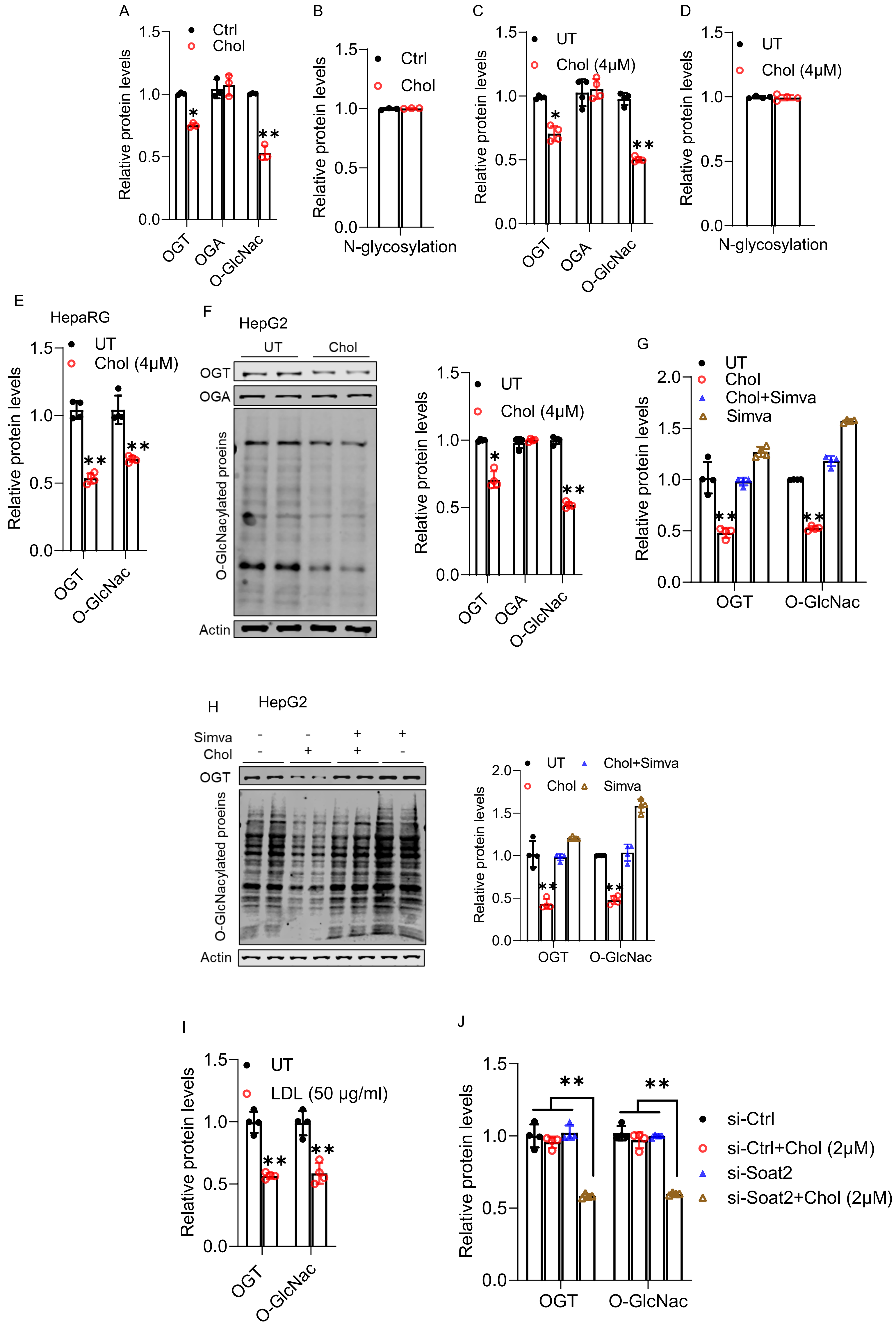

Suppl. Figure 4

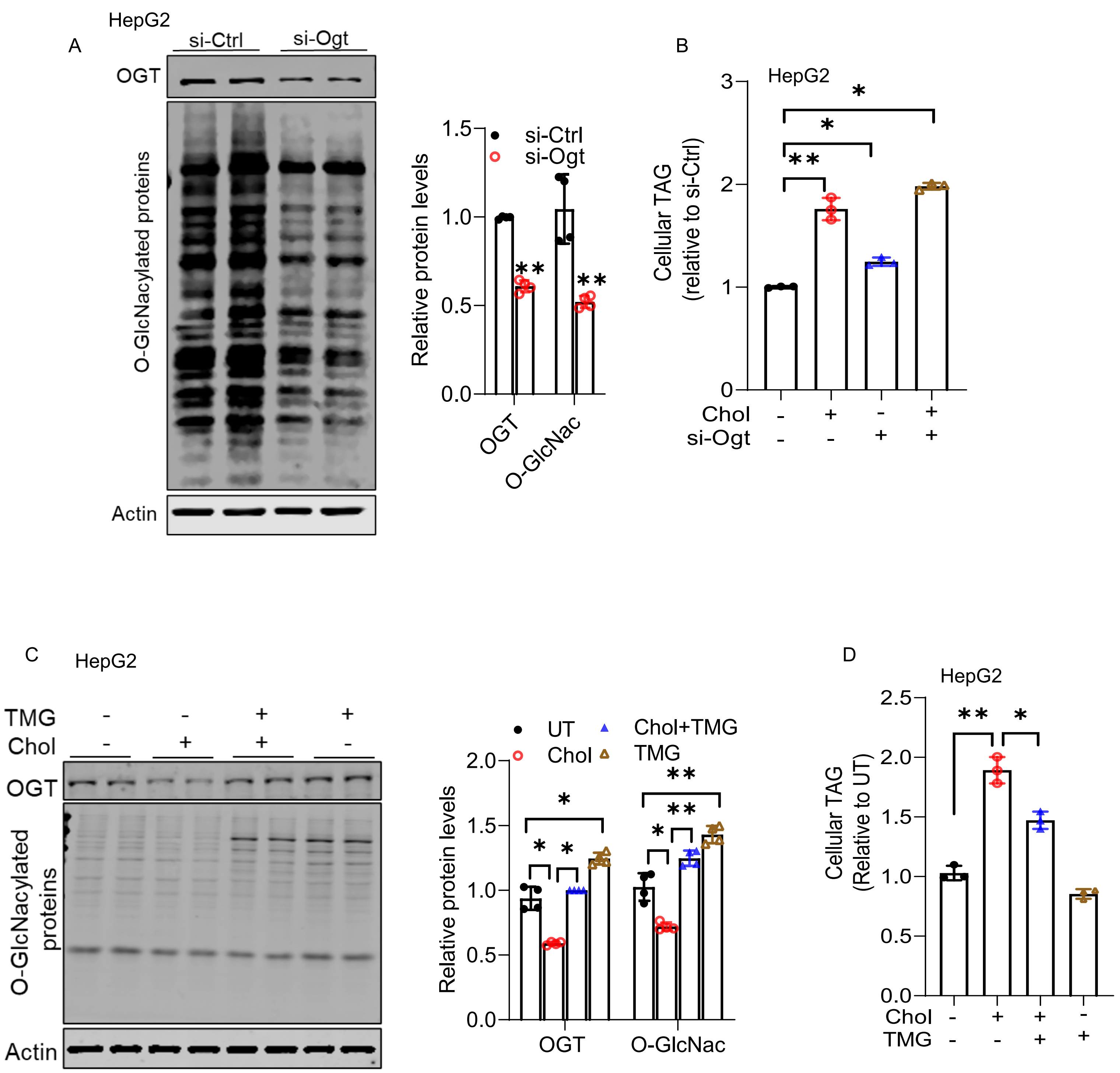

Suppl. Figure 5

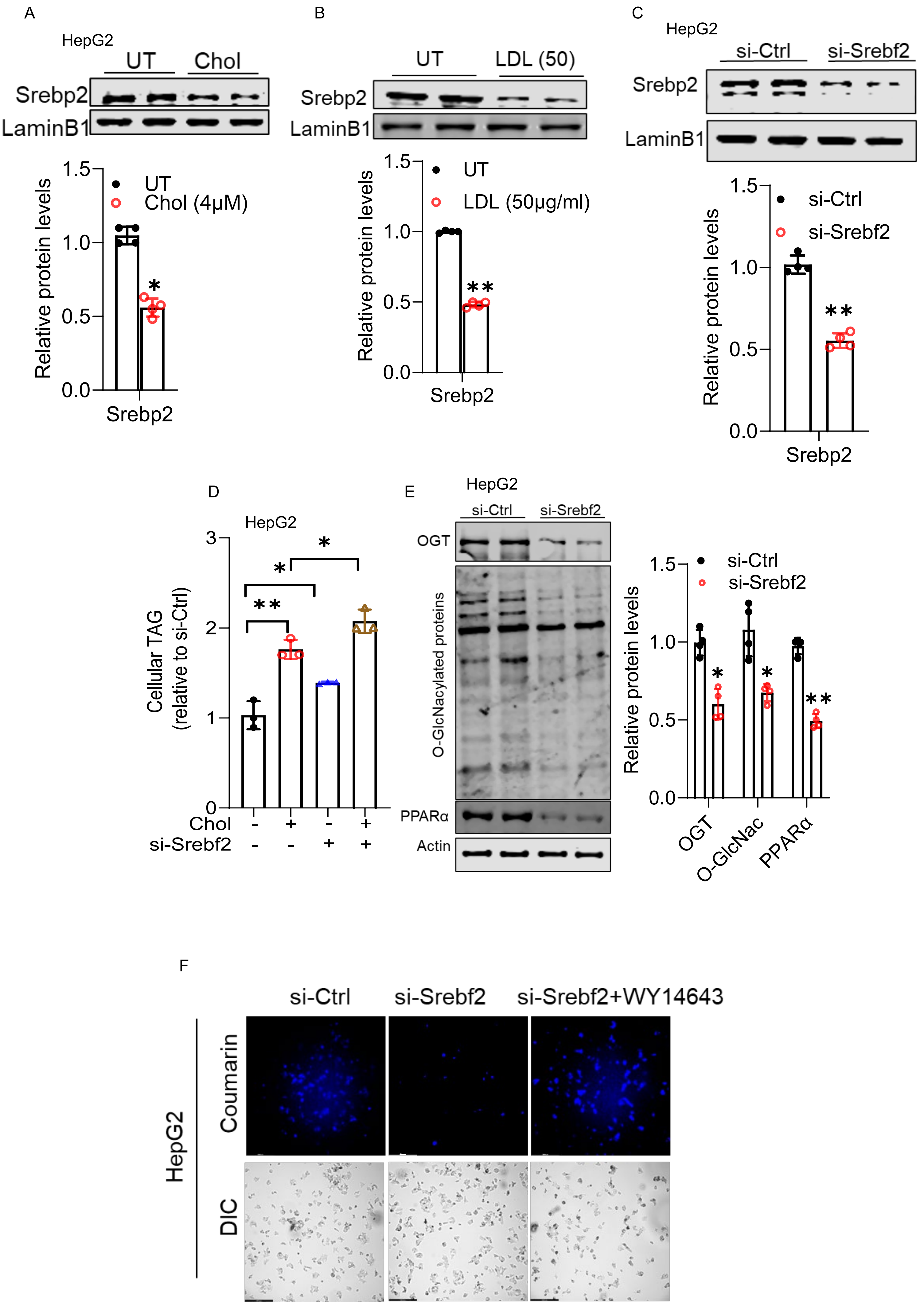

Supplement: Supplementary file 1 — Supplementary materials and methods, figures. [file ijbsv22p5615s1.pdf]
